# Effects of GWAS-Associated Genetic Variants on lncRNAs within IBD and T1D Candidate Loci

**DOI:** 10.1371/journal.pone.0105723

**Published:** 2014-08-21

**Authors:** Aashiq H. Mirza, Simranjeet Kaur, Caroline A. Brorsson, Flemming Pociot

**Affiliations:** 1 Copenhagen Diabetes Research Center (CPH-DIRECT), Department of Pediatrics E, Herlev University Hospital, Herlev, Denmark; 2 Faculty of Health and Medical Sciences, University of Copenhagen, Copenhagen, Denmark; 3 Center for non-coding RNA in Technology and Health, University of Copenhagen, Copenhagen, Denmark; National Cancer Institute, National Institutes of Health, United States of America

## Abstract

Long non-coding RNAs are a new class of non-coding RNAs that are at the crosshairs in many human diseases such as cancers, cardiovascular disorders, inflammatory and autoimmune disease like Inflammatory Bowel Disease (IBD) and Type 1 Diabetes (T1D). Nearly 90% of the phenotype-associated single-nucleotide polymorphisms (SNPs) identified by genome-wide association studies (GWAS) lie outside of the protein coding regions, and map to the non-coding intervals. However, the relationship between phenotype-associated loci and the non-coding regions including the long non-coding RNAs (lncRNAs) is poorly understood. Here, we systemically identified all annotated IBD and T1D loci-associated lncRNAs, and mapped nominally significant GWAS/ImmunoChip SNPs for IBD and T1D within these lncRNAs. Additionally, we identified tissue-specific *cis*-eQTLs, and strong linkage disequilibrium (LD) signals associated with these SNPs. We explored sequence and structure based attributes of these lncRNAs, and also predicted the structural effects of mapped SNPs within them. We also identified lncRNAs in IBD and T1D that are under recent positive selection. Our analysis identified putative lncRNA secondary structure-disruptive SNPs within and in close proximity (+/−5 kb flanking regions) of IBD and T1D loci-associated candidate genes, suggesting that these RNA conformation-altering polymorphisms might be associated with diseased-phenotype. Disruption of lncRNA secondary structure due to presence of GWAS SNPs provides valuable information that could be potentially useful for future structure-function studies on lncRNAs.

## Introduction

Long non-coding RNAs (lncRNAs) are recently discovered class of non-coding RNAs that are 200 nucleotides or longer in transcript length, are similar to protein-coding genes and are sometimes transcribed as whole or partial antisense transcripts to coding genes [1], [2]. LncRNA genes are poorly conserved in sequence across different species and do not contain any conserved motifs [3]–[5]. Like other RNA species, lncRNAs also form secondary structures that play critical roles in functional mechanisms [6]. In recent years, lncRNAs have emerged as important regulatory players of gene expression. Increasing number of studies have implicated lncRNAs in a wide range of biological and cellular processes including development [7], localization [8], alternative splicing [9], chromatin remodeling [10], cell cycle [11], survival [12], migration [13] and metabolism [6]. Importantly, lncRNAs are known to modulate gene expression by both *cis* and *trans* acting manner, and broadly wield their effect by direct interaction with the chromatin-modifying proteins and transcription factors, promoter inactivation by binding to basal transcription factors and activation of an accessory protein, [1], [14]–[18]. Furthermore, lncRNAs can also regulate gene expression at post-transcriptional level by binding to specific miRNAs and subsequently preventing these miRNAs from binding to their target mRNA transcripts [19]. Antisense lncRNA transcripts have also been shown to control the transcription of protein-coding genes in *cis* [20], [21].

The role of lncRNAs in various human diseases has recently been collated and described elsewhere [22], [23]. Accumulating body of evidence has linked mutations, alterations in the primary structure, secondary structure, and expression levels of lncRNAs to a number of human pathologies including autoimmune diseases, cancers and neurodegenerative diseases [13], [15], [16], [22], [24]–[27]. More recently, an elegant and comprehensive study explored the strand specific transcriptome of human pancreatic islets and beta cells, and identified 1128 islet specific lncRNA genes involved in beta cell differentiation and maturation [28]. Additionally, in recent years, genome-wide association studies (GWAS) have been salutary in identifying a large number of disease predisposing single nucleotide polymorphisms (SNPs) particularly in the autoimmune diseases [29]–[31]. Surprisingly, only a fraction of these identified variations are located within the protein-coding genes and a majority of these SNPs map to the non-coding intervals [32]–[34] including lncRNAs, and many of these genetic variations are likely to have a role in gene regulation [35], [36]. A powerful method to elucidate the genetic component underlying altered gene expression is mapping of expression quantitative trait loci (eQTL) [37]. eQTLs that map, and regulate nearby genes are referred to as *cis*-eQTLs. In contrast, eQTLs that map and regulate distant genes or on a different chromosome, are referred to as *trans*-eQTLs [37].

SNP-induced changes may affect transcription-factor binding sequences, translational efficiency and *trans* regulators such as lncRNAs and miRNAs. Recent evidence suggests that the disease-associated SNPs located within the regulatory regions of non-coding RNAs could potentially perturb the structural motifs and disrupt their function, and thereby lead to disease [38]. Nevertheless, underlying molecular mechanisms by which these genetic variations within the functional motifs of non-coding RNAs potentially affect their regulatory domains and cause abrogation of molecular interactions remains to be elucidated. Underpinning such mechanisms could be advantageous in unraveling the functional roles of lncRNAs and their-associated SNPs in disease context.

Inflammatory bowel disease (IBD) and type 1 diabetes (T1D) are immune-mediated diseases that share common susceptibility pathways and genes. Comparative analysis of susceptibility loci between different immune-mediated disorders has delineated important insights into their common underlying genetic architecture. Both, IBD and T1D share multiple loci, however, often with contrasting ramifications. For example, a misssense SNP (R620W, rs2476601) in protein tyrosine phosphatase non-receptor type 22 (PTPN22) has been shown to be associated with several autoimmune diseases including T1D, rheumatoid arthritis and Crohn's disease (CD) but with opposite directions of association [39]. However, functional and structural consequences of autoimmune disease associated genetic variations located within the IBD and T1D loci-associated lncRNAs have largely remained an elusive and unaddressed question.

**Figure 1:**
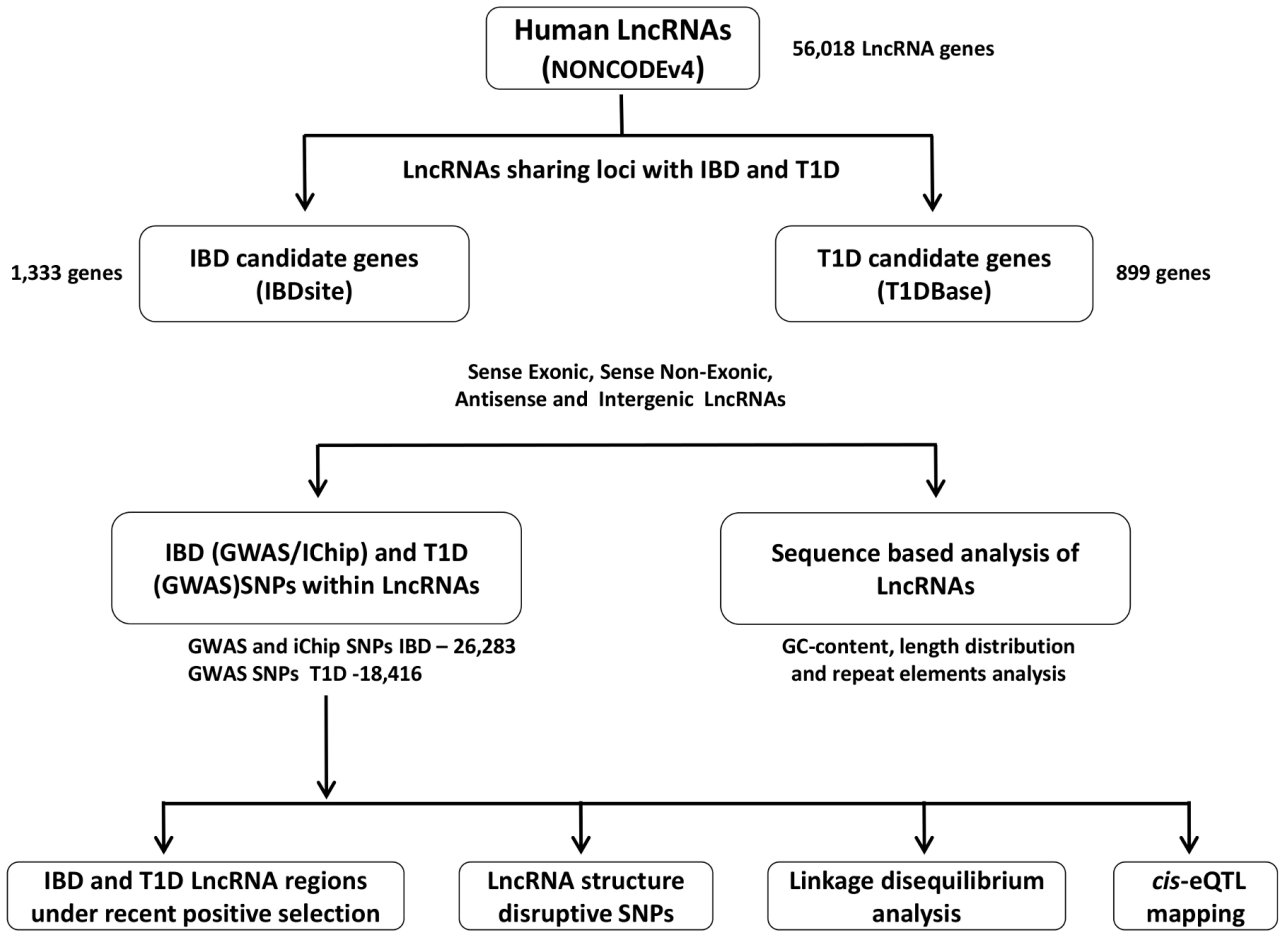
Schematic outline of the analysis pipeline. The summary of the steps involved in this study. All human lncRNAs (sense exonic, sense non-exonic, antisense and intergenic) located within and in close proximity (5 kb up/downstream) of the IBD and T1D candidate genes were identified. Based on the above described workflow, we predicted potential lncRNA secondary structure-disruptive GWAS SNPs within the IBD and T1D loci-associated lncRNAs. cis-eQTL signals were identified for these lncRNAs and linkage disequilibrium analysis was also explored for selected SNPs. We exploited HapMap data to identify candidate lncRNAs under recent positive selection.

In the present study, we conducted systematic search for all annotated IBD and T1D loci-associated lncRNAs, and demarcated their sequence and structural based characteristics. We identified structure-disruptive SNPs within the linkage disequilibrium (LD) blocks defined for the GWAS/ImmunoChip loci and predicted their effect on the lncRNA secondary structure. Furthermore, we also identified distinct tissue-specific expression patterns, *cis*-eQTLs signals, ENCODE based regulatory features and evidence for recent positive selection for the IBD and T1D loci-associated lncRNAs. These findings suggest that single genetic risk variants located within non-coding regions could play regulatory roles and possibly alter function of lncRNAs by modulating their expression and spatial physiognomy which in turn could profoundly affect neighboring (*cis*) or distal (*trans*) candidate genes. The overall pipeline employed for this study is outlined in Figure 1. Although, we restricted our study to IBD and T1D loci, this approach can be further extended for other autoimmune disorders.

## Results

### IBD and T1D loci-associated lncRNAs

**Figure 2:**
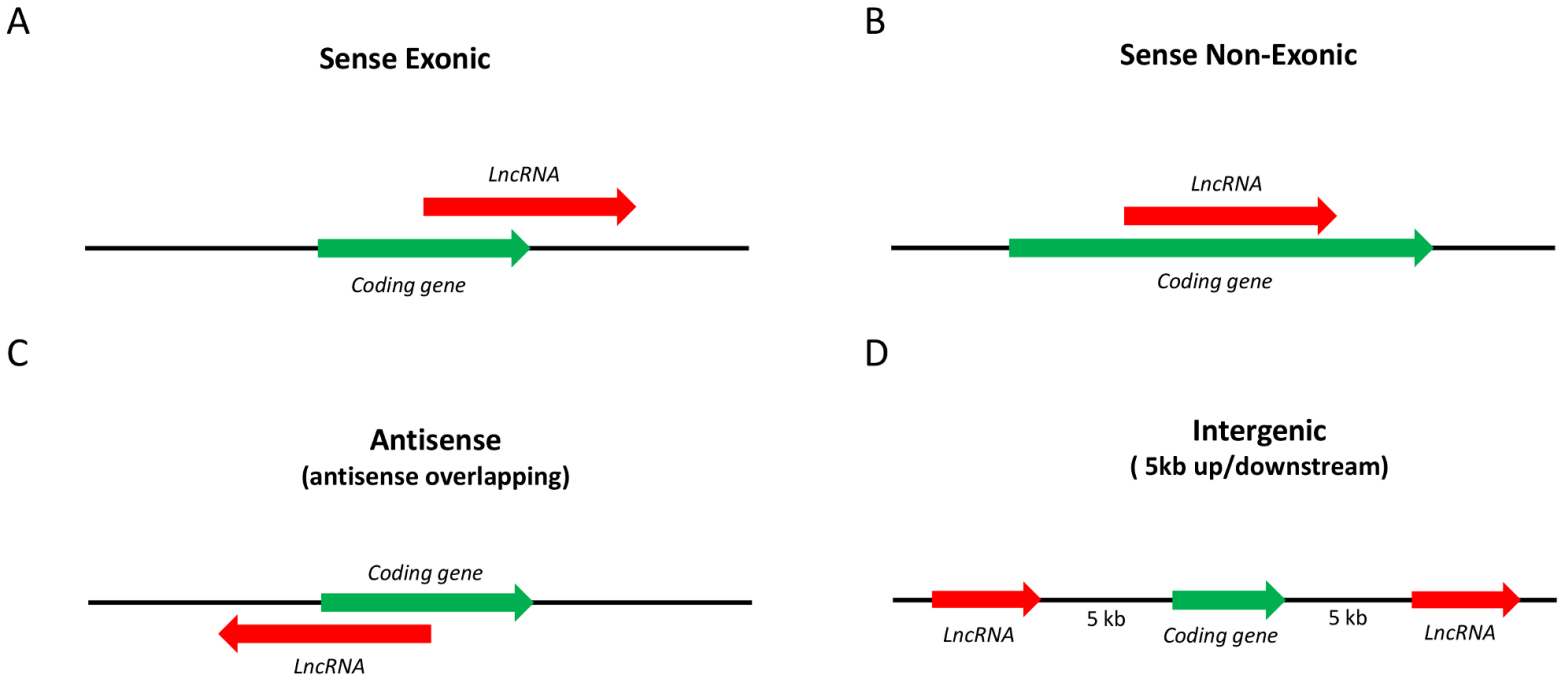
Mapped lncRNA intervals within IBD and T1D susceptibility loci genes. Categorization of the genomic association of the lncRNAs to the IBD and T1D loci-associated genes, as sense exonic (A), sense non-exonic (B), antisense (C), and intergenic (D).

Majority of lncRNAs are transcribed as complex interlaced networks sharing genomic sequences within number of different intersecting coding and non-coding transcripts in sense and antisense directions [40]. On dissecting the IBD and T1D loci datasets, we found 3665 lncRNA genes intersect 1168 IBD candidate genes (Table 1). Based on strand orientation (Figure 2), 1440 lncRNA genes were found to be antisense to 750 IBD candidate genes. Also, 2133 lncRNA genes intersected 1004 IBD candidate genes with 100% *cis*-overlap on the same strand. 2245 lncRNA genes intersected 1038 IBD candidate genes with an overlap of at least one nucleotide on the same strand. In case of the T1D loci, 762 lncRNA genes intersected 660 T1D candidate genes. Likewise, when taking strand orientation into account, 317 lncRNA genes were found to be antisense of 297 T1D candidate genes. Furthermore, 611 lncRNA genes intersected 473 T1D candidate genes with 100% *cis*-overlap on the same strand. We also observed 690 lncRNA genes intersecting 579 T1D candidate genes on the same strand with an overlap of at least one nucleotide.

**Table 1:** IBD and T1D candidate gene associated lncRNAs.

	IBD		T1D	
Category	LncRNA genes	IBD genes	LncRNA genes	T1D genes
**Exonic/Non-Exonic and Antisense lncRNAs within IBD and T1D candidate genes**				
Sense intersecting	2245	1038	690	579
Sense 100% overlapping	2133	1004	611	473
Antisense intersecting	1440	750	317	297
**Total**	**3665**	**1168**	**762**	**660**
**Intergenic lncRNAs in 5 kb up/down-stream proximity of IBD and T1D candidate genes**				
Sense	395	339	344	364
Antisense	752	624	383	422
**Total**	**1131**	**778**	**537**	**588**
**Total mapped Exonic/Non-Exonic, Antisense and Intergenic lncRNA genes**				
**Overall Total**	**4272**	**1231**	**816**	**761**

IBD and T1D candidate gene associated lncRNAs located in and around the (+/−) 5 kb up/downstream of start and end site of each candidate gene. Number of exonic/non-exonic, antisense lncRNAs are described for both IBD and T1D.

For lncRNAs located in the proximity of IBD and T1D candidate genes, we screened 5 kb up/down-stream flanking regions to identify intergenic lncRNAs associated with IBD and T1D loci. Regardless of the strand orientation, we identified 1131 and 537 intergenic lncRNA genes 5 kb up/down-stream of 778 IBD and 588 T1D candidate genes, respectively (Table 1). While considering strand orientation, 752 and 383 antisense lncRNA genes mapped to 624 IBD and 422 T1D candidate genes, respectively. Also, 395 and 344 lncRNA genes mapped to the same strand to 339 IBD and 364 T1D candidate genes, respectively with an overlap of at least one nucleotide.

In total, we identified 4272 and 816 lncRNA genes in and around 5 kb up/down-stream proximity of 1231 IBD and 761 T1D candidate genes, respectively. Furthermore, following the Noncodev4 classification criteria for lncRNA categories based on their genomic location in relation to all protein-coding genes, we identified 1692 sense exonic, 579 sense non-exonic, 1345 antisense and 767 intergenic IBD loci-associated lncRNAs. While in case of the T1D loci-associated lncRNAs, we identified 381 sense exonic, 53 sense non-exonic, 188 antisense and 225 intergenic lncRNAs.

### Sequence analysis of IBD and T1D loci-associated lncRNAs

The sequence features such as GC content and repetitive elements of coding and non-coding genes are well known attributes coupled to their biological functions. In vertebrates including humans, GC content is known to vary significantly between different genomic regions [41]. Several studies have established that GC content is strongly associated with various genomic features like gene density, recombination rate, and distribution of repetitive elements within the human genome [42]. The average length distribution (log transformed) of all lncRNA genes is shown in density plot (Figure S1). Our sequence analysis of IBD and T1D loci-associated lncRNAs revealed that lncRNAs associated with the T1D loci are shorter in length (average length  = 11,784) as compared to both IBD loci-associated lncRNAs (average length  = 23,285) as well as to total lncRNAs (average length  = 15,929) (Figure S2). The overall length distribution of IBD loci-associated lncRNAs varied significantly compared to the total lncRNAs (p-value < 10e-6, Welch two sample t-test). For IBD and T1D candidate genes, the average GC content was found to be 42% and 47% respectively. The average GC content of all known lncRNA genes was found to be 42%. IBD and T1D loci-associated lncRNA genes had an average GC content of 43.5% and 48% respectively (Figure S3). Our results showed significantly higher GC content in T1D loci-associated lncRNA genes (p-value < 10e-6, Welch two sample t-test) as compared to the background (total lncRNAs).

Furthermore, we also analyzed the relative abundance of various classes of repeat elements within all annotated human lncRNAs. Approximately, 81% of the total lncRNA genes (45,880 lncRNA genes), were found to harbor repeat elements. It was observed that the interspersed repeat families, i.e. SINEs, LINEs, LTRs and DNA elements were the most abundant repeat classes within the lncRNA genes and constituted 85% of the total repeats (Figure S4 and S5). Overall percentage of interspersed repeat families observed within these lncRNA genes was 34%, 27%, 13% and 9% for SINEs, LINEs, LTRs and DNA elements, respectively. SINE repeat family was the most abundant repeat family in the lncRNAs, which is also the most abundant repeat family in human genome after LINEs. Interestingly, we observed very few low complexity regions and simple repeats within the lncRNA genes. We also compared the distribution of repeats within the IBD and T1D loci-associated lncRNAs. Our analysis revealed that in IBD loci-associated lncRNA genes, almost 85% of the lncRNAs harbored repeat elements. The interspersed repeat elements (35% SINEs, 28% LINEs, 11% LTRs and 10% DNA elements) constituted 84% of the total repeats found within the IBD loci-associated lncRNAs. In case of T1D loci-associated lncRNA genes, 79% of the lncRNA genes harbored repeat elements and interspersed repeat elements constituted 85% of the total repeats (39% SINEs, 26% LINEs, 10% LTRs and 10% DNA elements). The interspersed repeat element distributions for both IBD and T1D loci-associated lncRNAs were found to significantly different (p-value < 10e-6, Chi-square goodness of fit test) than the background (total lncRNAs). In case of SINEs within the T1D loci-associated lncRNAs, the absolute value of standardized residuals was found to be 12 (z score >1.96). These results suggest significant enrichment of SINEs in T1D loci-associated lncRNAs.

### GWAS/ImmunoChip SNPs within IBD and T1D loci-associated lncRNAs

Since vast majority of GWAS signals map to the non-coding regions of the genome where they are known to play many regulatory roles [33], [34], we mapped all the nominally associated IBD and T1D SNPs within the IBD and T1D loci-associated lncRNAs. Overall, 26,283 GWAS/ImmunoChip SNPs were retrieved for IBD and 18,416 GWAS SNPs for T1D loci. All these SNPs were first mapped to all the human lncRNA genes, and then to the IBD and T1D loci-associated lncRNA genes. In case of all the human lncRNA genes, 7893 IBD SNPs were found to be present within 2523 lncRNA genes, whereas, 5273 T1D SNPs were found to be present within 2235 lncRNA genes. For IBD loci-associated lncRNA genes, 2063 SNPs were found to be present within 468 lncRNA genes. For T1D loci-associated lncRNA genes, 1045 SNPs were found to be present within 247 lncRNA genes. Within the shared IBD and T1D-associated lncRNA genes, we found 44 common SNPs. The SNPs that mapped within the IBD and T1D loci-associated lncRNAs were selected for further analysis to predict their potential to disrupt the lncRNA secondary structures.

### LncRNA structure-disruptive GWAS/ImmunoChip SNPs in IBD and T1D

**Figure 3:**
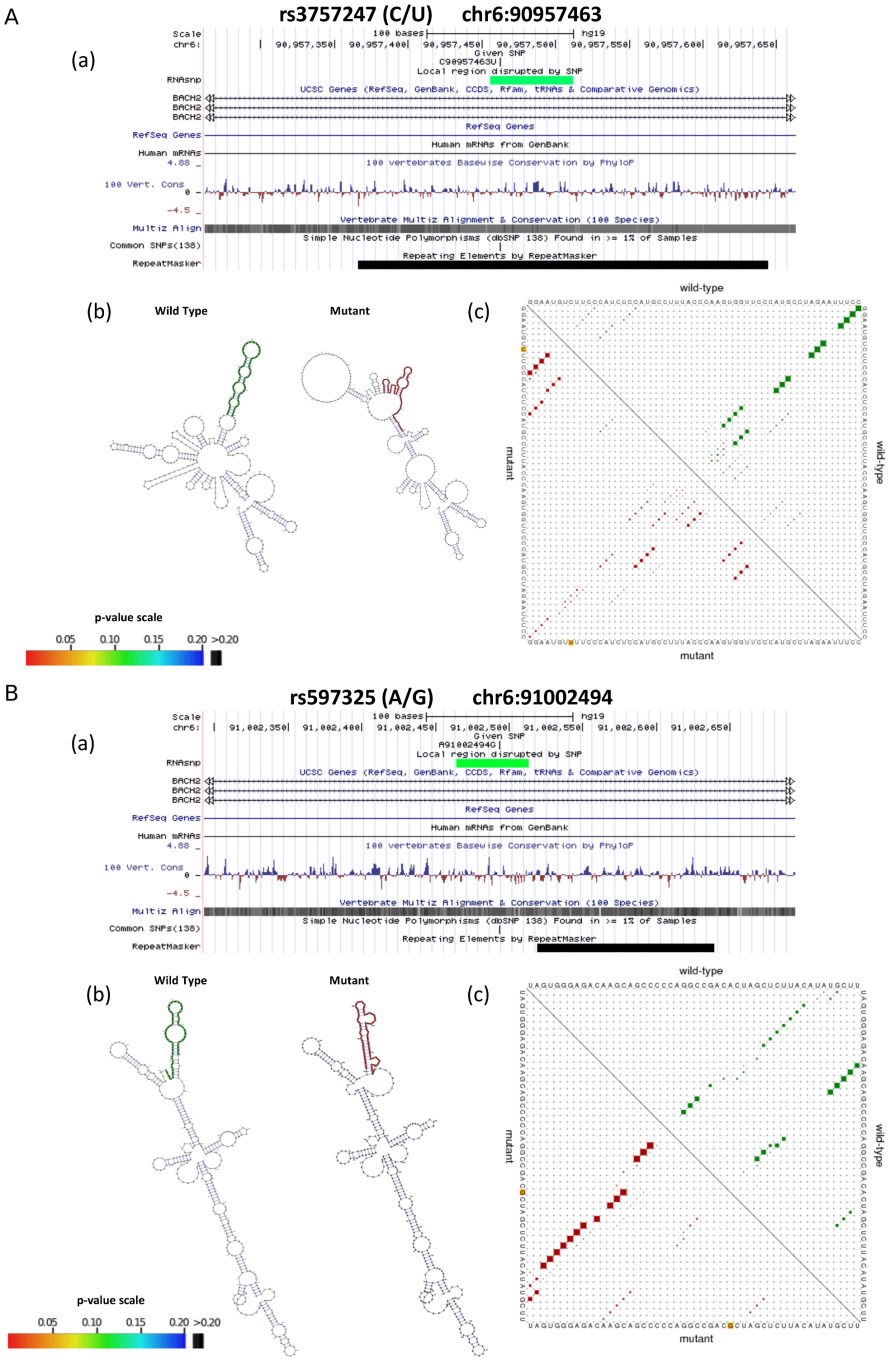
Structure disruption of lncRNA *NONHSAG044354*-associated with *BACH2* (implicated in both IBD and T1D) by GWAS SNPs rs3757247 and rs597325. The structure-disruptive effects of SNPs rs3757247 (A) and rs597325 (B) located in lncRNA *NONHSAG044354* associated with candidate gene *BACH2*. (a) UCSC genome browser view showing the location of the predicted local region disrupted by the SNP. The color of predicted local region (green in this case) is based on the RNAsnp p-value (0.129 and 0.120 for rs3757247 and rs597325 respectively). (b) Minimum free energy structures (MFE) of the global wild-type and mutant sequences displaying the secondary structure and the local region affected by the SNP position, colored green (wild-type) and red (mutant) (c) Dot plot representing the base pair probabilities of wild type and mutant RNA sequences corresponding to the predicted local region by RNAsnp. The upper and lower triangle of the matrix represents the base pair probabilities of wild-type (green) and mutant (red), respectively.

Studies have demonstrated that SNPs residing in and around key regulatory region of the lncRNA genes in the genome are known to be significantly associated with the increased susceptibility to sundry diseases [36]. We focused on the impact of GWAS/ImmunoChip SNPs within IBD and T1D loci-associated lncRNAs, and identified number of SNPs with significant propensity to disrupt secondary structure of the loci-associated lncRNAs. The SNP-induced structural perturbations within the lncRNAs could be disruptive for its molecular functions and therefore, could possibly contribute towards the disease phenotype. From our analysis, we present a list of 362 and 178 structure-disruptive SNPs in the IBD and T1D loci-associated lncRNAs respectively that were found to be causing significant secondary structure changes in their-associated lncRNAs with empirical p-value <0.2 (Table S1). For example, 2 SNPs (rs3757247 and rs597325) perturbs the secondary structure of the sense exonic lncRNA *NONHSAG044354* associated with candidate gene *BACH2* (Figure 3).

### Common structure-disruptive SNPs between IBD and T1D loci-associated lncRNAs

**Figure 4:**
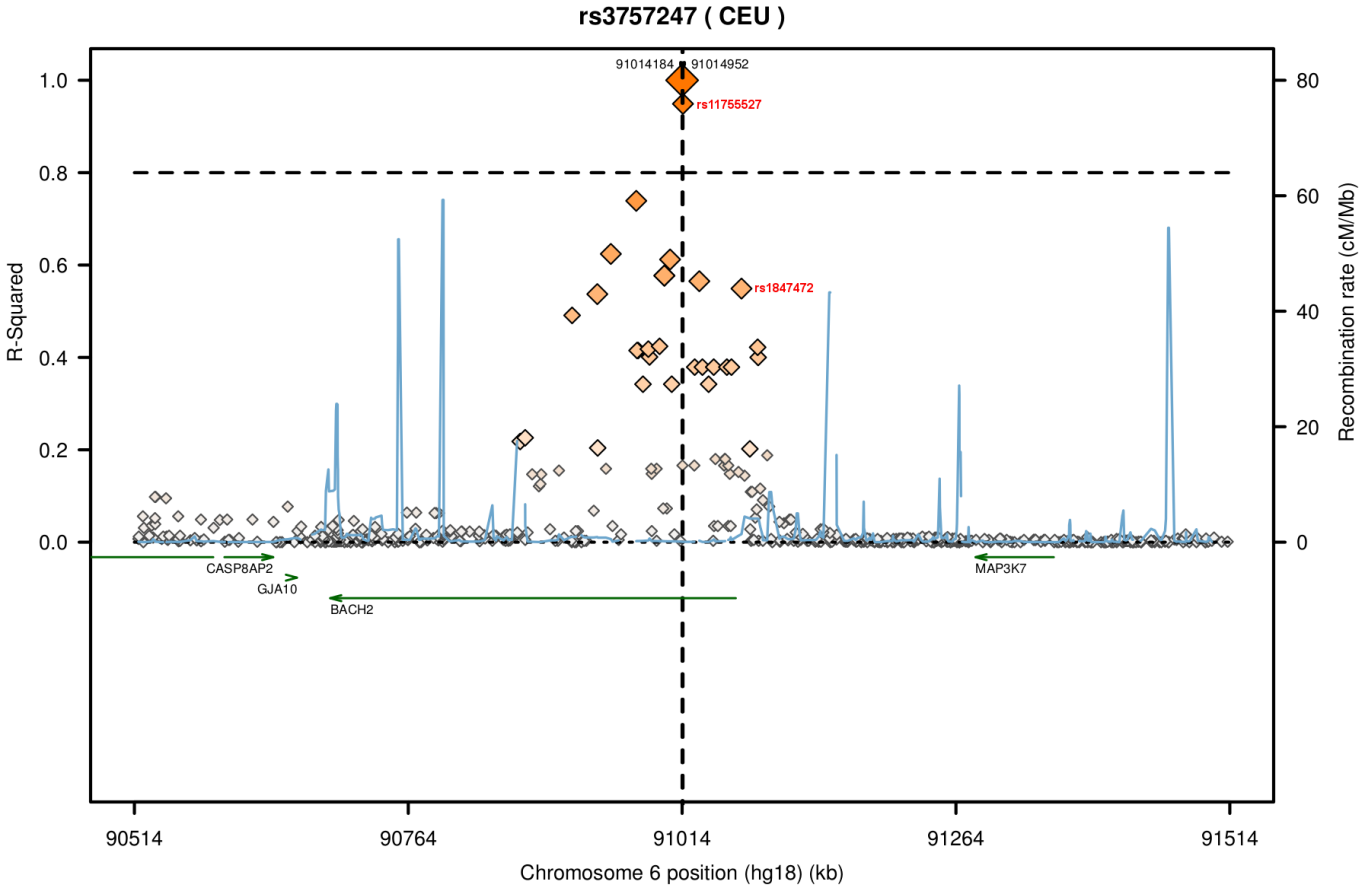
Regional LD plot for SNP rs3757247 associated with IBD and T1D loci. The regional LD plot for SNP rs3757247 was calculated using HapMap3 (release 2) in SNAP tool. SNPs are represented as diamonds and the brightness of each SNP is proportional to the r^2^ threshold value for that SNP. SNPs rs11755527 and rs1847472 had an r2 value of 0.949 (T1D p-value_meta_ 5.38e-08, Barrett et al 2009 [52]) and 0.565 (IBD p-value_ichip_ 1.19e-04, IBD p-value_meta_ 1.10e-09, Jostins et al 2012 [71]) respectively (shown in red).

Considering the commonality of the associated risk loci between IBD and T1D diseases, we searched for common structure-disruptive SNPs shared between their loci-associated lncRNAs. Indeed, we found seven structure-disruptive SNPs (rs5763746, rs1476514, rs41176, rs41158, rs3757247, rs597325 and rs602662) to be common and located in the same locus of IBD and T1D-associated lncRNAs (Table 2). Four of these SNPs (rs5763746, rs1476514, rs41176 and rs41158) were located within a single antisense lncRNA *NONHSAG033653*. Interestingly, lncRNA *NONHSAG033653* is in close proximity of the *HORMAD2* (22q12.2) candidate gene, which has been implicated in both IBD and T1D. SNP rs41158 was found to be located within the T1D candidate gene *MTMR3*. An index SNP rs602662 was found to be present within antisense lncRNA *NONHSAG026183* and candidate gene *FUT2* (19q13.33) [71]. Two SNPs rs3757247 and rs597325 were located within lncRNA *NONHSAG044354* and *BACH2* gene (6q15), an important candidate gene in both IBD and T1D (Figure 3). Investigating the pattern of LD across the *BACH2* locus revealed that the structure-disruptive SNP rs3757247 significantly correlated (r^2^ = 0.949) with the T1D risk SNP rs11755527, as well as in strong correlation (r^2^ = 0.565) with the IBD risk SNP rs1847472 (Figure 4).

**Table 2:** Common structure-disruptive SNPs within IBD and T1D loci-associated lncRNAs.

Structure-disruptive SNPs common to IBD and T1D loci-associated lncRNAs				GWAS p-values		Shared lncRNA genes	Candidate genes	
	Chr	Position	SNP ID	IBD (Jostins et al 2012 [71])	T1D (Barrett et al 2009 [52])		IBD	T1D
1	chr22	30450660	rs5763746	4.07e-03	9.51e-03	NONHSAG033653(-)	HORMAD2 (+)	HORMAD2(+), MTMR3(+), CTA-85E5.10(−), CNN2P1(−)
2	chr22	30433822	rs1476514	4.53e-03	7.41e-05			
3	chr22	30432403	rs41176	2.78e-04	6.48e-13			
4	chr22	30405983	rs41158	1.82e-03	9.99e-09			
5	chr6	90957463	rs3757247	5.44e-03	8.05e-08	NONHSAG044354(-)	BACH2(−)	RP3-453I5.2(−), BACH2(−)
6	chr6	91002494	rs597325	2.26e-03	6.36e-10			
7	chr6	49206985	rs602662	1.87e-03	1.93e-05	NONHSAG026183(-)	FUT2(+)	FUT2(+)

Four SNPs common between an IBD and T1D loci-associated antisense lncRNA were in close proximity of a protein coding gene *HORMAD2*, which has been implicated in both IBD and T1D and three other T1D candidate genes *MTMR3* (protein coding gene), *CTA-85E5.10* (processed transcript), *CNN2P1* (pseudogene). Two SNPs rs3757247 and rs597325 were located within lncRNA *NONHSAG044354* and a protein coding gene *BACH2* and a pseudogene *RP3-453I5.2*. SNP rs602662 was found to be present within antisense lncRNA *NONHSAG026183* and protein coding gene *FUT2*. Strand is displayed in brackets for lncRNAs and their-associated IBD/T1D candidate genes.

### ENCODE annotation and *cis*-eQTL mapping of structure-disruptive SNPs in IBD and T1D loci-associated lncRNAs

Disease associated SNPs are highly enriched within the ENCODE-defined non-coding functional element regions. In many cases, these SNPs are known to have regulatory functions, and some are associated with bona fide eQTLs [37]. We retrieved ENCODE annotation for 362 and 178 structure-disruptive SNPs in IBD and T1D loci-associated lncRNAs using RegulomeDB database [43]. We found, 254 out of 362 and 143 out of 178 structure-disruptive SNPs in IBD and T1D loci-associated lncRNAs were associated with transcription factor (TF) binding, eQTLs, DNase peak and therefore were likely to affect the binding.

**Figure 5:**
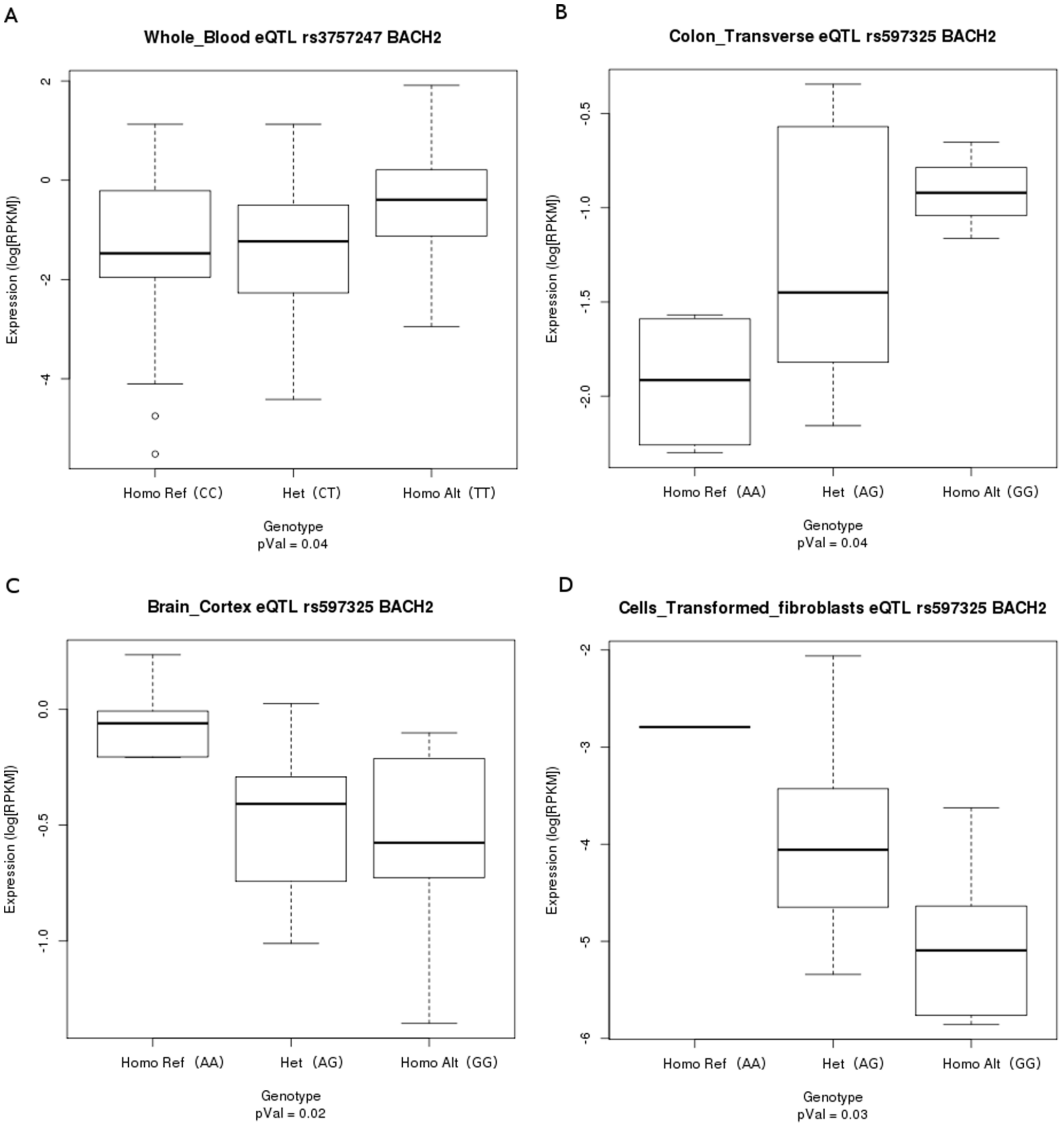
*cis*-eQTLs and gene-SNP associations for rs3757247 and rs597325 with *BACH2* candidate gene. (A) The gene SNP association for rs3757247 and *BACH2* candidate gene in whole blood. (B, C, D) The gene SNP association for rs597325 and *BACH2* in colon transverse, brain cortex and cell line fibroblasts. The gene SNP associations are calculated using linear regression model using Genotype Tissue Expression Portal (GTEx) in the selected tissues with more than 80 samples using a *cis* window of +/−1 MB around the transcription start site (TSS) at significance level of 0.05. For each gene SNP association plot, p-values are displayed at the bottom.

We retrieved pre-computed significant *cis*-eQTLs from 9 tissues (adipose subcutaneous, artery tibial, heart left ventricle, lung, muscle skeletal, nerve tibial, skin exposed sun, thyroid and whole blood) tested in more than 80 samples using a *cis* window of +/−1 MB around the transcription start site (TSS) for the structure-disruptive SNPs in IBD and T1D loci-associated lncRNAs using GTEx [44]. Out of 362 structure-disruptive SNPs from IBD loci-associated lncRNAs, only 94 SNPs had associated *cis*-eQTLs (Table S1). In case of 178 structure-disruptive SNPs in T1D loci-associated lncRNAs, only 28 SNPs had *cis*-eQTLs. We also examined the gene-SNP association patterns for the structure-disruptive SNPs within IBD and T1D loci-associated lncRNAs, and found significant associations for these SNPs. For example, we observed significant association of the common structure-disruptive SNP (rs3757247) for both IBD and T1D loci-associated lncRNA *NONHSAG044354* with IBD and T1D candidate gene *BACH2* only in the whole blood (Figure 5). We also observed significant tissue-specific *cis*-eQTL signals associated with *P4HA2*, *P4HA2-AS1*, and *AC063976.6* genes in thyroid, *SLC22A5* gene in whole blood, lung and skin sun exposed and *AC034220.3* gene in whole blood (Table S1).

### IBD and T1D loci-associated lncRNAs with structure-disruptive SNPs under recent selection pressure

A number of studies report that many genes exhibit very strong signals of recent selection in favor of new alleles. These regions of recent positive selection indicate the presence of genetic variants that are a source of significant phenotypic variation [45]. Indeed, it is known that many of these variants affect complex phenotypes of clinical relevance [46]–[48]. Based on selective screening of Haplotter and HapMap [45], [49] datasets, we identified structure-disruptive SNPs located within the IBD and T1D loci-associated lncRNAs that are under very recent selection in favor of new alleles, and have not yet reached fixation. Out of 362 structure-disruptive SNPs in IBD loci-associated lncRNAs, 12 SNPs (present within 14 IBD loci-associated lncRNAs) were found to be under recent positive selection in CEU, ASI, ASN and YRI populations respectively. In case of 178 structure-disruptive SNPs in T1D loci-associated lncRNAs, 13 SNPs (present within 14 T1D loci-associated lncRNAs) in CEU and YRI populations were found to be under recent positive selection (Table S1). The presence of structure disruptive SNPs under recent positive selection within IBD and T1D loci-associated lncRNAs, provides evidence for the functional roles of these variants.

Based on RegulomeDB score, *cis*-eQTLs, recent positive selection and RNAsnp p-value (p-value < = 0.2), we subsequently ranked 362 and 178 structure-disruptive SNPs of IBD and T1D loci-associated lncRNAs respectively (Table 3 and S1). Structure disruption caused by the top ranked SNPs rs2227319 and rs243327 within the IBD and T1D loci-associated lncRNAs respectively along with their *cis*-eQTLs is described in Figure S6 and S7.

**Table 3:** Structure-disruptive SNPs and their-associated lncRNAs.

Structure-disruptive SNPs	IBD	T1D
Structure-disruptive SNPs (P-val < = 0.2)	362	178
LncRNA genes harboring structure-disruptive SNPs	192	102
Total IBD/T1D candidate genes harboring structure-disruptive SNPs	124	94
Protein-coding genes harboring structure-disruptive SNPs	118	63
Structure-disruptive SNPs with annotated ENCODE data using RegulomeDB	259	143
Structure-disruptive SNPs having *cis*-eQTLs	94	28
Structure-disruptive SNPs under recent positive selection	12	13
Candidate lncRNAs under recent positive selection harboring structure-disruptive SNPs	14	14

Total numbers of structure-disruptive SNPs are reported after ranking based on RegulomeDB score, *cis*-eQTLs and recent positive selection.

### Expression profiles of IBD and T1D loci-associated lncRNAs

**Figure 6:**
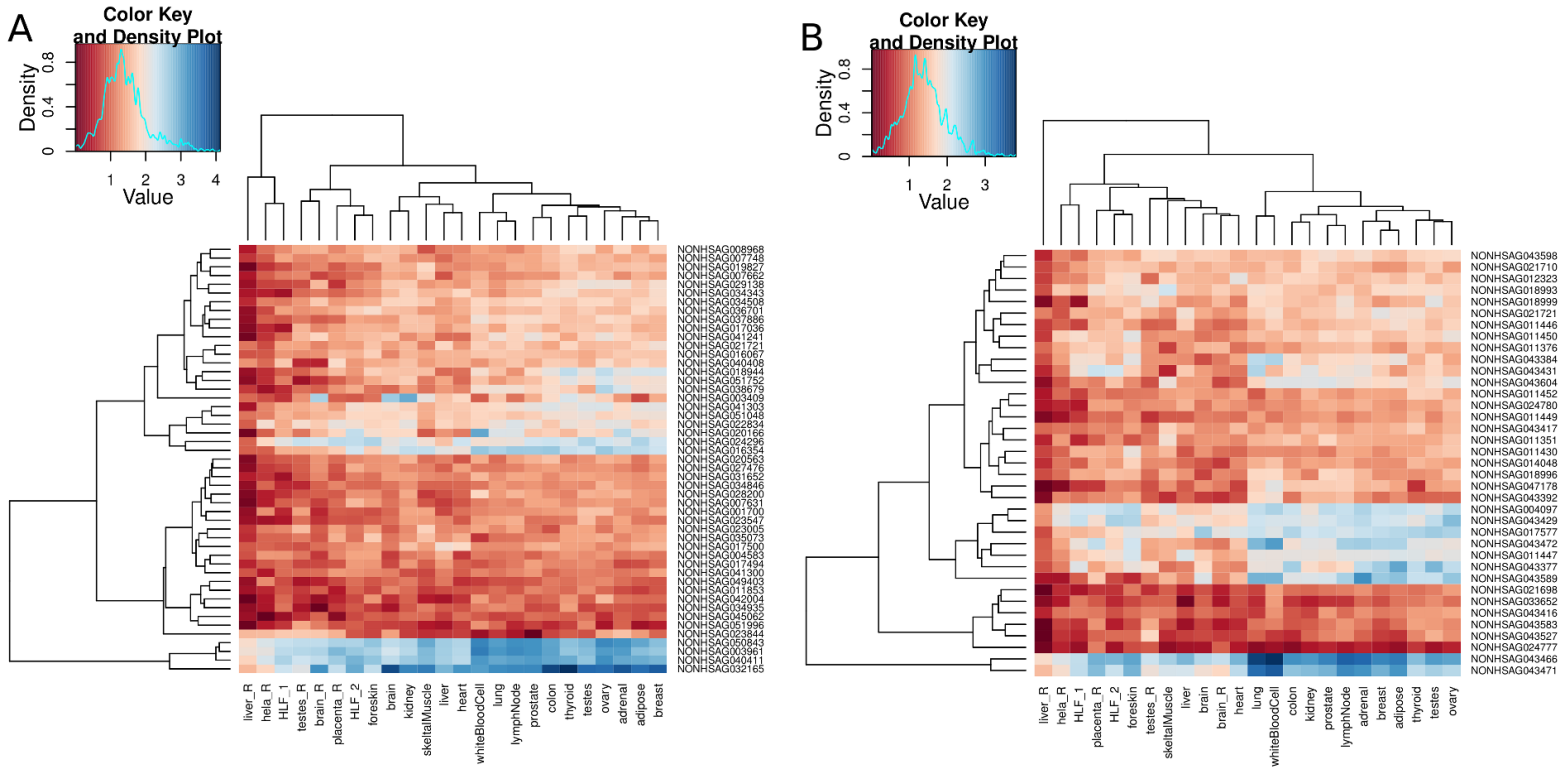
Expression of antisense IBD and T1D loci-associated lncRNAs. Expression profiles for (A) 49 antisense IBD loci-associated lncRNAs and (B) 37 antisense T1D loci-associated lncRNAs expressed across all the HBM tissues. The FPKM threshold of >1 was used and the values were log10 transformed.

**Figure 7:**
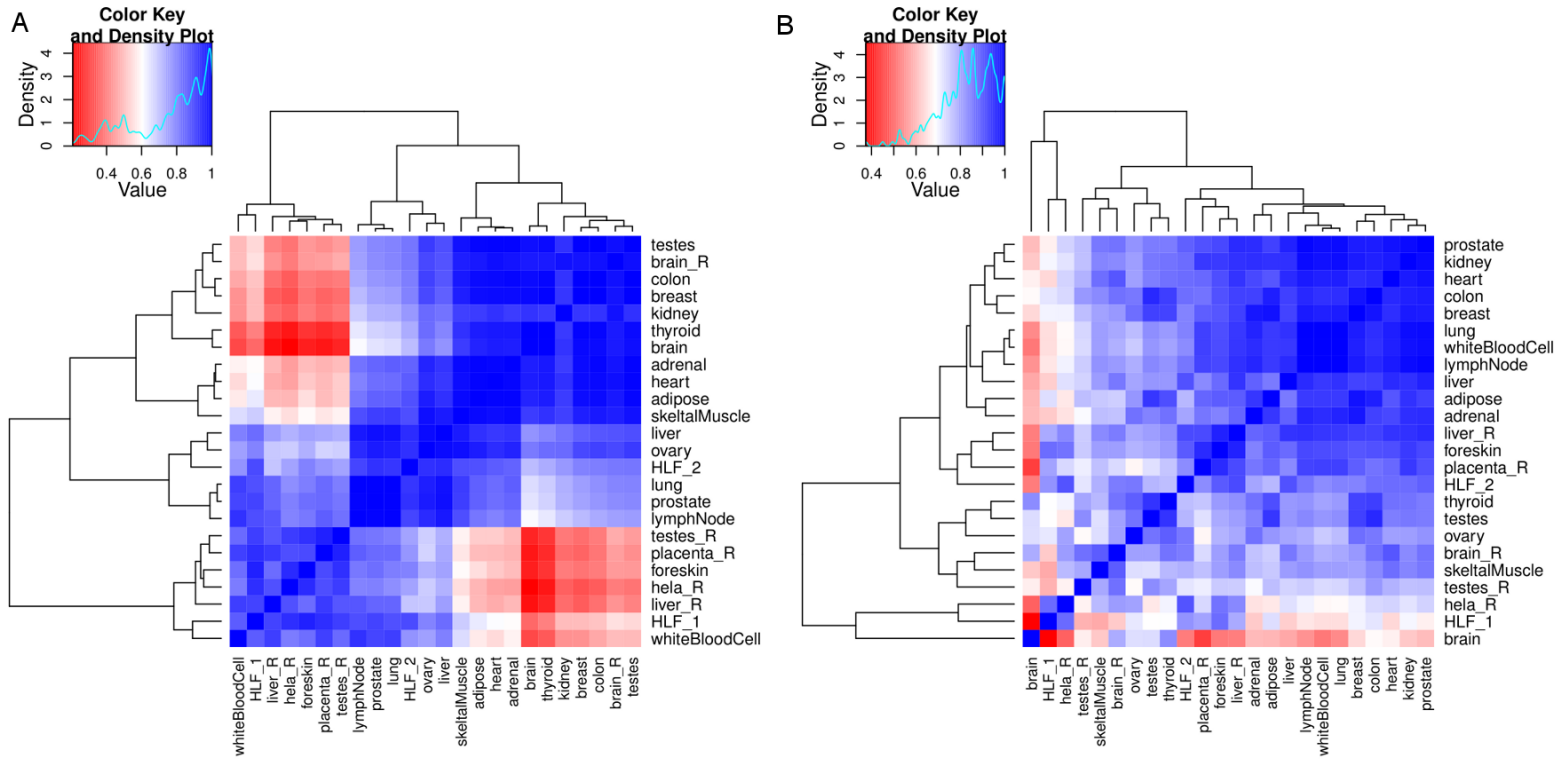
Correlations of expression for antisense IBD and T1D loci-associated lncRNAs. Spearman correlations for (A) antisense IBD and (B) antisense T1D loci-associated lncRNAs expressed across all the HBM tissues. The FPKM threshold of >1 was used and the values were log10 transformed.

In-depth systematic analysis of lncRNA expression in multiple human tissues has revealed that lncRNAs are usually lower expressed than protein-coding genes, and exhibit distinct tissue and developmental stage-specific expression patterns [50]. In our datasets, we explored the expression profiles of IBD and T1D loci-associated lncRNAs using Human BodyMap (HBM) data. We observed a number of IBD and T1D loci-associated lncRNAs expressed across all the HBM tissues using a threshold of >1 FPKM (Figure 6, S8 and S9). Based on this threshold, 251 out of 4272 and 79 out of 816 IBD and T1D loci- associated lncRNAs respectively were found to be expressed across all the HBM. Whereas, for the sense exonic/non-exonic lncRNAs mapping 100% within the IBD and T1D candidate genes, 155 out of 2133 and 68 out of 611 IBD and T1D loci-associated lncRNAs respectively were found to be expressed across all HBM tissues (Figure S8 and S9). In case of the antisense IBD and T1D loci-associated lncRNAs, only 49 out of 1440 and 37 out of 317 were expressed across all tissues, respectively (Figure 6). We also calculated spearman correlations for the lncRNAs expressed across all the tissues to identify tissues that clusters together based on highly similar expression patterns. For the antisense lncRNAs, we observed diverse patterns of expression for IBD and T1D loci-associated lncRNAs (Figure 7). In case of the sense exonic/non-exonic and intergenic lncRNAs, we observed significant differences in expression patterns across the tissues for IBD and T1D loci-associated lncRNAs (Figure S10 and S11). In addition, for the IBD loci-associated lncRNAs, on average 72% of lncRNAs were not detected in any tissue, while in case of the T1D loci-associated lncRNAs, 61% of the lncRNAs were not detected in any of the tissues at an FPKM threshold of >1 (Table S2).

**Figure 8:**
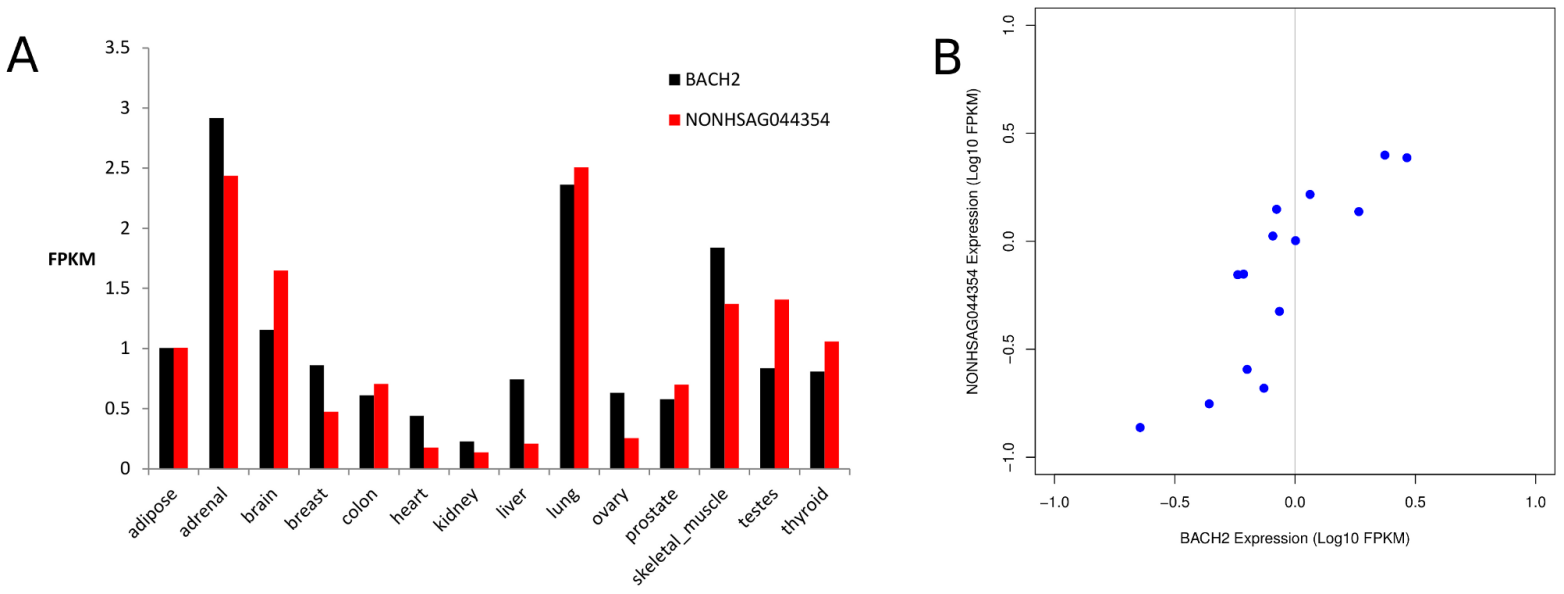
Comparison of expression levels of *NONHSAG044354* with its host gene (*BACH2*) in 14 tissues. (A)Tissue-specific gene expression profile of *BACH2* candidate gene and *NONHSAG044354* associated lncRNA across 14 tissues based on Human Body Map (HBM) data. (B) Sense exonic lncRNA *NONHSAG044354* positively correlating (r_s_ = 0.85) with *BACH2* candidate gene. Protein coding mRNA expression is plotted on the x-axis and lncRNA expression is shown on the y-axis both on log10 FPKM for 14 HBM tissues.

Furthermore, from our analysis, we observed a noticeable tissue-specific differential expression patterns in lncRNAs and their associated protein-coding genes across an array of 14 tissues. We observed differential expression profile of IBD and T1D loci-associated lncRNA *NONHSAG044354* and its associated candidate gene *BACH2* in most of the HBM tissues except in adipose, colon, and prostrate (Figure 8A). We also found tissue-specific differential expression for two other common IBD and T1D candidate genes *FUT2* and *HORMAD2* and their associated lncRNAs (Figure S12A and 12C). In addition, we observed positive spearman correlations for *BACH2*, *FUT2* and *HORMAD2* candidate genes with their associated lncRNAs (Figure 8B, S12B and S12D). Sense exonic lncRNA *NONHSAG044354* positively correlated (r_s_ = 0.85) with its associated candidate gene *BACH2* (Figure 8B). However, in the case of intergenic antisense lncRNA *NONHSAG033653* we observed a weaker positive correlation (r_s_ = 0.67) with its associated candidate gene *HORMAD2* (Figure S12B). Whereas, antisense lncRNA *NONHSAG026183* highly correlated (r_s_ = 0.95) with its associated candidate gene *FUT2* (Figure S12D). These data are in concordance with the recent findings of GENCODEv7 lncRNA catalog study in which similar specific enrichment of positive correlations of lncRNAs intersecting protein coding exons in antisense orientation with the mRNA host was also reported.

## Discussion

Genome-wide association studies (GWAS) have provided an excellent platform in pursuit of identifying common risk variants underlying many polygenic diseases [32], with unprecedented success especially in several autoimmune diseases [33]. Studies have suggested that comparative meta-analysis approach is an excellent tool for dissecting the autoimmune diseases risk loci, and it could help gain valuable insights into understanding the functional roles of the genes that are shared between these diseases, and uncover novel disease-loci relationships [51]–[53]. More than 50% of IBD susceptibility loci overlap with other inflammatory and autoimmune diseases including T1D. Most of the risk variants map to the regulatory non-coding regions of the genome including lncRNAs - which have recently emerged as key players in regulating fundamental cellular processes. LncRNAs are known to act as decoys and forestall the access of DNA-binding proteins such as transcription factors to the DNA. A class of distinctively expressed lincRNAs can make cell-type-specific “flexible scaffolds” and, these scaffolds in turn interact with the regulatory protein complexes and alter cell-type-specific gene expression programs [54].

Furthermore, recent studies have revealed significant similarities between lncRNAs and 3′ untranslated regions (3′ UTRs) in terms of their structural features and sequence composition [55]. LncRNAs and 3′UTRs exhibit lower GC content of 42% and 43% respectively as compared to the protein-coding RNAs (51.8%). The lower GC content in lncRNAs and 3′UTR sequences indicate that they might contain fewer stably base-paired structures making their primary sequence more accessible for interactions with cellular factors [55]. However, in our analysis, we found GC content of T1D loci- associated lncRNAs to be comparatively higher (48%) than the IBD loci-associated lncRNAs (43.5%) and total NONCODEv4 human lncRNAs (42%). Several studies have reported association of the GC content with various intrinsic genomic features such as distribution of repeat elements [42]. Interestingly, in case of T1D loci-associated lncRNAs, we observed over-representation of SINE elements as compared to the IBD loci-associated lncRNAs and all human lncRNAs. Collectively, we found approximately 82% of human lncRNAs harbor repetitive elements, and out of these around 92% harbor interspersed repeat elements. SINE elements such as Alu's have been implicated in the gene and miRNA regulation. Alu's within the 3′UTR have been found to be involved in Staufen1 (STAU1) mediated mRNA decay (SMD) [56]. Base-pairing between AluJo (100 nt) in the 3′-UTR of SERPINE1 gene and AluSx (300 nt) in 3′UTR of FLJ21870 gene with another Alu repeat located within a cytoplasmic polyadenylated lncRNA (AF087999) forms RNA-RNA duplexes that are targets for SMD [56]. Notably, the over-representation of Alu repeat elements within the T1D loci-associated lncRNAs suggest that these lncRNAs might be involved in similar regulatory functions based on RNA-RNA interactions.

Emerging lines of evidence suggests that expression dysregulation and large- and small-scale mutations in primary sequence of lncRNAs are strongly linked with phenotype changes and disease susceptibility. Genetic mutations are transmitted to the transcriptome during the lncRNA transcription events and could possibly perturb the lncRNA functions particularly if such mutations are located within the embedded regulatory motifs of lncRNAs. Indeed, various studies have established that genetic variations present within ncRNAs could potentially alter their functions predominantly by inducing changes in their folding patterns, secondary structure stability and affect expression. For example, multiple SNPs within 5′ and 3′ untranslated regions (UTRs) of the gene altered the mRNA structural ensemble of the-associated genes for six disease-states (hyperferritinemia cataract syndrome, beta-thalassemia, cartilage-hair hypoplasia, retinoblastoma, chronic obstructive pulmonary disease (COPD), and hypertension) [38]. Furthermore, specific GWAS SNPs in and around an antisense lncRNA ANRIL have been shown to alter the transcription and processing of ANRIL transcripts which are associated with increased susceptibility to coronary disease, intracranial aneurysm, type 2 diabetes, as well as several tumor types [24], [57], [58]. Therefore, exploring structural impact of these genetic variations within lncRNAs may provide additional insights and evidence regarding their functionality. Indeed, our analysis also identified highly significant structure-disruptive SNPs within IBD and T1D loci-associated lncRNAs that might be related with altered transcription levels of the lncRNAs and their associated protein coding genes.

Genetic variations can strongly affect the gene expression phenotype and various studies have also reported tissue-specific eQTLs [59], [60]. Recently, a number of disease- or trait- associated SNPs were found to be tissue-dependent lincRNA *cis*-eQTLs [36]. We also identified tissue-specific lncRNA *cis*-eQTLs associated with structure-disruptive SNPs. For example, in case of sense lncRNA *NONHSAG044354* associated with IBD and T1D candidate gene *BACH2*, we observed significant tissue-specific *cis*-eQTL signal but only in whole blood based on gene SNP association for the structure-disruptive SNP rs3757247 and *BACH2*. The propensity of variation in gene expression patterns observed between and within species is strongly determined by the adaptive changes in the gene regulation mechanisms [61]. Indeed, eQTL signals frequently map to the regions of the human genome that have undergone recent positive selection [62]. Interestingly, from our analysis we also identified a number of lncRNA *cis*-eQTLs under recent positive selection. For example, the antisense lncRNA *NONHSAG043608* associated with T1D candidate genes *SYNGAP1* and *ZBTB9*, we found signals for recent positive selection in YRI population and significant tissue-specific *cis*-eQTL associated with *HLA-DPB1* gene in thyroid tissue (Table S1). Whereas, in case of the antisense *NONHSAG041519* and sense *NONHSAG041520* lncRNAs associated with IBD candidate genes *SLC22A4* and *SLC22A5*,we found strong signals for recent positive selection in CEU population (Table S1).

Gene expression is a tightly controlled phenomenon and is differentially regulated across tissues and cell types. In case of lincRNAs, studies have demonstrated their spatiotemporal specific expression [3], [63], [64]. Expression analysis of IBD and T1D candidate genes and their associated lncRNAs using HBM dataset revealed diverse levels of tissue-specific expression patterns across all the tissues. LncRNAs intersecting exons of protein coding genes in sense and antisense orientations are known to be enriched for positive correlations with their mRNA host [50]. We also observed strong positive correlations for lncRNAs intersecting IBD and T1D candidate genes. For example, the IBD and T1D loci-associated lncRNAs *NONHSAG044354* and *NONHSAG026183* intersecting candidate genes *BACH2* and *FUT2* respectively showed strong positive correlations with their host mRNA expression levels (Figure 8 and S12). These findings suggest that expression of the host mRNA could be regulated by their intersecting sense and antisense lncRNAs. *BACH2* is one of the common candidate gene implicated in multiple inflammatory diseases including IBD and T1D and recently, it has been shown to regulate the CD4+ T-cell differentiation that in turn averts the inflammatory disease by maintaining the balance between tolerance and immunity [65]. In our analysis we observed a strong LD signal between BACH2 associated lncRNA (*NONHSAG044354*) structure-disruptive SNP rs3757247 and T1D risk SNP rs11755527 and also with IBD risk SNP rs1847472 in CEU population (Figure 4). SNP rs11755527 has also been reported to be strongly associated with the thyroid peroxidase autoantibodies (TPOA) [66]. In a recent study, disease associated SNP pairs that are in high LD were found to form structure stabilizing haplotypes in UTRs of the human genome [67]. Notably, two of the SNPs from our ranked list of structure-disruptive SNPs in IBD loci-associated lncRNAs (Table S1) were also reported to alter the ensemble of RNA structures in the above study. Both of these SNPs rs1050088 (*DAG1* 3′UTR variant) and rs2836723 (lincRNA *AF064858* exon variant) are known IBD associated variants (GWAS p-value 7.34e-12 and 2.94e-05 [71]). Taken together, these data further suggest that specific pairs of lncRNA associated SNPs that are in high LD could form RNA structure-stabilizing haplotypes altering the ensemble of structures adopted by the mRNA. Nevertheless, genetic variation driven lncRNA structure changes alone may not be the only underlying mechanism manifesting disease phenotype, but rather an outcome from an array of cumulative molecular aberrations including loss of regulatory binding sites coupled with expression dysregulation that together defines the phenotype.

## Materials and Methods

### Data retrieval

We retrieved protein coding and non-coding genes within the IBD and T1D regions (referred as candidate genes in this study). These IBD and T1D regions are based on genome-wide association studies (p<5e-08) or have attained significant association in a candidate gene study. Inflammatory bowel disease (IBD) candidate genes were retrieved from the IBDsite database [68]. IBDsite database contains data related to the biomolecular mechanisms-associated with the onset of IBD and it also hosts the human and bacterial information related to Crohn's disease (CD) and Ulcerative colitis (UC). A total of 1432 IBD human candidate genes were retrieved and filtered to remove their isoforms and antisense transcripts. After filtering, the IBD dataset included 1333 IBD candidate genes. T1D regions were retrieved from T1Dbase (version 4.15) [69]. T1Dbase is a web based resource focused on the genetics and genomics of type 1 diabetes susceptibility (T1D) in mouse, rat and human. In total, 899 T1D candidate genes were retrieved from the T1Dbase. Human long non-coding RNAs (lncRNAs) were retrieved from NONCODEv4 database [70]. NONCODE is an integrated database of all types of noncoding RNAs (except tRNAs and rRNAs) obtained from various sources. The current database contains 95,135 (56,018 lncRNA genes) human annotated lncRNA transcripts. Combined GWAS and ImmunoChip SNPs for IBD were retrieved from the International Inflammatory Bowel Disease Genetics Consortium (IIBDGC) database {http://www.ibdgenetics.org} [71]. This dataset is based on meta-analysis of GWAS datasets after imputation to the HapMap3 reference set, and replicated in the ImmunoChip data any SNPs with p<0.01. Meta-analyzed GWAS SNPs for T1D were retrieved from T1DGC [52]. In case of the T1D GWAS SNPs, a cut off based on p-value<0.01 was used to filter nominally significant SNPs. Since coordinates of both datasets, were based on human genome build (GrCh36/hg18), hence all the SNP coordinates were converted to the current build of the human genome (GrCh37/hg19).

### IBD and T1D loci-associated lncRNAs and GWAS/ImmunoChip SNPs

Human lncRNA gene coordinates from NONCODEv4 database were intersected with candidate gene coordinates of IBD and T1D loci using intersectBed feature from the BedTools suite [72] to identify lncRNA genes located within IBD and T1D loci. LncRNAs present within the proximity of 5kb up/down-stream of the IBD and T1D candidate genes were also identified. miRNA genes located within mapped IBD and T1D loci-associated lncRNAs, and the GWAS/ImmunoChip and GWAS SNPs for IBD and T1D loci were mapped to the IBD and T1D loci-associated lncRNAs using the intersectBed. In our dataset, based on the genomic association of lncRNAs to the protein coding genes, we categorized lncRNAs as sense exonic, sense non-exonic, antisense, and intergenic (Figure 2).

### Sequence analysis of IBD and T1D loci–associated lncRNAs

IBD and T1D loci-associated lncRNAs were subjected to sequence analysis such as comparison of their length distribution, GC content and presence of various repeat elements. Repeatmasker version 4.0.2 [73] was used to compare the distribution and repeat classes within the IBD and T1D loci–associated lncRNAs using RepBase library dated 2013-04-22.

### Structure-disruptive SNPs in IBD and T1D loci-associated sense exonic/non-exonic and intergenic lncRNAs

We employed RNAsnp [74] to predict the structural effects of GWAS SNPs within the IBD and T1D loci-associated lncRNAs. RNAsnp focuses on the local regions of maximal structural change between wild-type and mutant and the mutation effects are quantified in terms of empirical p-values. Moreover, RNAsnp uses extensive pre-computed tables of the distribution of SNP effects as function of sequence length, GC content and SNP position. For our analysis we used mode1 option of the RNAsnp which employs global folding and a folding window of +/−200 nucleotides around the SNP position to calculate base pairing probability matrices for wild type and mutant subsequences. The difference between wild type and mutant base pair probability for the local regions is measured using Euclidean distance with the corresponding p-values. The local region detected with maximum structural change with p-value less than 0.2 was considered as significant structural change. All the SNPs passing the p-value threshold of <0.2 were selected for further downstream analysis.

### eQTLs, ENCODE annotation, LD, and tissue-specific expression of structure-disruptive SNPs in IBD and T1D loci-associated lncRNAs

To retrieve *cis*-eQTLs for structure-disruptive SNPs in IBD and T1D loci-associated lncRNAs, we used Genotype-Tissue Expression project resource (GTEx) (http://www.broadinstitute.org/gtex/) [44]. For a query SNP, GTEx provides pre-computed significant *cis*-eQTLs from 9 tissues (adipose subcutaneous, artery tibial, heart left ventricle, lung, muscle skeletal, nerve tibial, skin sun exposed, thyroid and whole blood) with more than 80 samples using a *cis* window of +/−1 MB around the transcription start site (TSS) [44]. We also calculated eQTLs in selected tissues based on gene-SNP associations using GTEx for all those structure-disruptive SNPs for which no pre-computed *cis*-eQTLs were available.

All regulatory features and ENCODE annotations for structure-disruptive SNPs in IBD and T1D loci-associated lncRNAs were retrieved from RegulomeDB database [43]. RegulomeDB annotates SNPs with known and predicted regulatory elements in intergenic regions of human genome using datasets from GEO [75], ENCODE project [76], and published literature.

Regional LD plots for selected SNPs were generated using SNP annotation and proxy search (SNAP) [77]. We selected HapMap3 (release 2) SNP data-set and a distance limit of 500 kb.

The tissue-specific expression profiles for all IBD and T1D candidate genes and their associated lncRNA genes were retrieved using Human BodyMap 2.0 data (ENA archive: ERP000546) across 16 human tissues and NONCODEv4 [70]. Expression values were expressed as fragments per kilobase of exon per million reads (FPKMs), which is a measure of gene expression normalized to size of the gene and RNA-seq library size. Expression values (FPKMs) were log10 transformed and graphically represented as heatmaps for IBD and T1D loci-associated lncRNAs using an FPKM threshold of >1 (Figure 6, S8 and S9).

### IBD and T1D loci-associated lncRNAs under recent positive selection

To identify loci-associated lncRNAs under recent positive selection, we focused on structure-disruptive SNPs present within the IBD and T1D loci-associated lncRNAs. We took leverage of Haplotter [45], a tool based on HapMap project data [49]. Haplotter is based on integrated Haplotype Score (iHS), a statistic used to detect evidence of recent positive selection at a locus, and covers iHS data for three populations, ASN (combined Japanese from Tokyo, Japan and Han Chinese from Beijing, China), CEU (Utah residents with Northern and Western European ancestry from the CEPH collection) and YRI (Yorubans from Ibadan, Nigeria). Only SNPs with extreme iHS scores (iHS > = 2.5 or iHS< = (−2.5)) were retrieved [45]. The IBD and T1D loci-associated lncRNAs that are under recent positive selection were identified based on the above described criteria.

## Supporting Information

Figure S1 **Log 10 transformed length distribution of Noncodev4 human lncRNA genes.**
(TIFF)

Figure S2 **Comparison of length distribution of IBD and T1D loci-associated lncRNA genes.** IBD and T1D loci-associated lncRNAs revealed significant differences in their average lengths as compared to the total lncRNAs.
(TIFF)

Figure S3 **Comparison of GC content of IBD and T1D loci-associated lncRNA genes.** Significantly higher GC content in T1D loci-associated lncRNA genes (p-value < 10e-6, Welch two sample t-test) was observed as compared to the background (total lncRNAs).
(TIFF)

Figure S4 **Distribution of repeat elements in Noncodev4 human lncRNA genes.**
(TIFF)

Figure S5 **Relative abundance of interspersed repeat elements within IBD and T1D loci-associated lncRNA genes.** Significant differences in the interspersed repeat element distributions for both IBD and T1D loci-associated lncRNAs were observed (p-value < 10e-6, Chi-square goodness of fit test) as compared to the background (total lncRNAs).
(TIFF)

Figure S6 **Top two structure-disruptive SNPs within IBD and T1D loci-associated lncRNAs (ranked based on RegulomeDB score and RNAsnp p-value).** (A) SNP rs2227319 (structure-disruptive SNP within IBD loci-associated lncRNA NONHSAG021725). (B) SNP rs243327 (structure-disruptive SNP within T1D loci-associated lncRNA NONHSAG018599).
(TIFF)

Figure S7 **cis-eQTLs for the top two structure-disruptive SNPs within IBD and T1D loci-associated lncRNAs (ranked based on RegulomeDB score and RNAsnp p-value).** (A) cis-eQTLs for structure-disruptive SNP rs2227319 within IBD loci-associated lncRNA NONHSAG021725. (B) cis-eQTLs for structure-disruptive SNP rs243327 within T1D loci-associated lncRNA NONHSAG018599. For each gene SNP association plot, p-values are displayed at the bottom. For a query SNP, GTEx provides pre-computed significant *cis*-eQTLs from 9 tissues (adipose subcutaneous, artery tibial, heart left ventricle, lung, muscle skeletal, nerve tibial, skin sun exposed, thyroid and whole blood) with more than 80 samples using a *cis* window of +/−1 MB around the transcription start site (TSS).
(TIFF)

Figure S8 **Expression levels for intergenic (A) and sense exonic/non-exonic (B) IBD loci-associated lncRNAs expressed across all HBM tissues (at FPKM threshold of >1).** The FPKM threshold of >1 was used and the values were log10 transformed.
(TIFF)

Figure S9 **Expression levels for intergenic (A) and sense exonic/non-exonic (B) T1D loci-associated lncRNAs expressed across all HBM tissues (at FPKM threshold of >1).** The FPKM threshold of >1 was used and the values were log10 transformed.
(TIFF)

Figure S10 **Correlation of expression for intergenic (A) and sense exonic/non-exonic (B) IBD loci-associated lncRNAs respectively expressed across all HBM tissues.** The FPKM threshold of >1 was used and the values were log10 transformed.
(TIFF)

Figure S11 **Correlation of expression for intergenic (A) and sense exonic/non-exonic (B) T1D loci-associated lncRNAs respectively expressed across all HBM tissues.** The FPKM threshold of >1 was used and the values were log10 transformed.
(TIFF)

Figure S12 **Tissue-specific gene expression profile of** ***HORMAD2*** **(A) and** ***FUT2*** **(C) candidate genes and their associated lncRNAs** ***NONHSAG033653*** **and** ***NONHSAG026183*** **across 14 tissues based on HBM data.** Spearman correlations were calculated for lncRNAs *NONHSAG033653* (B) and *NONHSAG026183* (D) with *HORMAD2* and *FUT2* candidate genes respectively. Protein coding mRNA expression is plotted on the x-axis and lncRNA expression is shown on the y-axis both on log10 FPKM for 14 HBM tissues.
(TIFF)

Table S1 **Structure-disruptive SNPs in IBD and T1D loci-associated lncRNAs ranked based on RegulomeDB, cis-eQTLs, recent positive selection and RNAsnp p-value.**
(XLSX)

Table S2 **LncRNA expression profile of IBD and T1D loci-associated lncRNAs.** Noncodev4 was used to retrieve expression data for sense exonic/non-exonic and antisense IBD and T1D loci-associated lncRNAs. Total number of expressed sense exonic/non-exonic and antisense lncRNAs was calculated using FPKM threshold >1.
(DOCX)
