# Supplementary figures and images for: Effects of GWAS-Associated Genetic Variants on lncRNAs within IBD and T1D Candidate Loci
Source: PLoS One. 2014 Aug 21;9(8):e105723. doi: 10.1371/journal.pone.0105723 (PMC4140826; doi:10.1371/journal.pone.0105723)

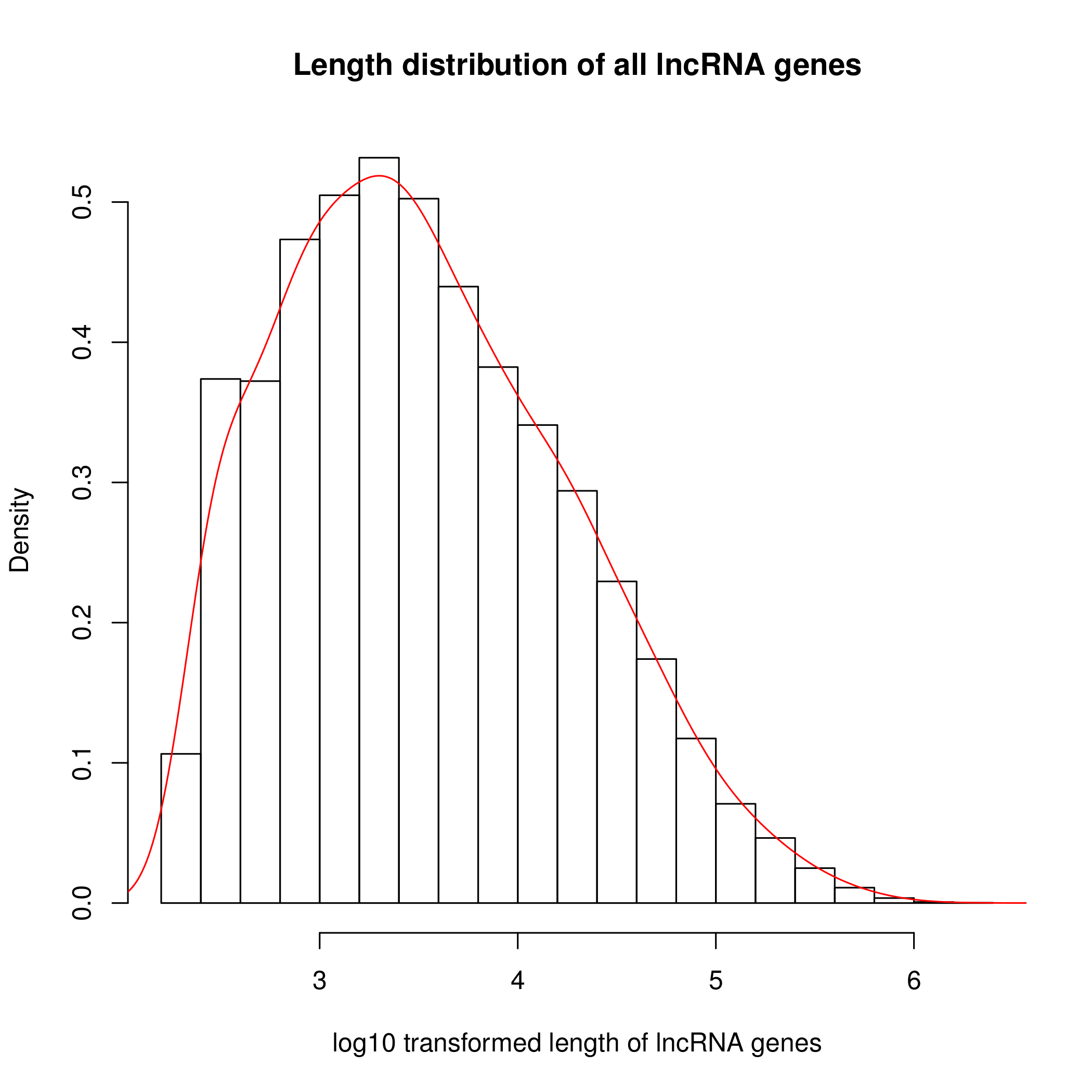

Supplement: Figure S1 — Log 10 transformed length distribution of Noncodev4 human lncRNA genes. (TIFF) [file pone.0105723.s001.tiff]

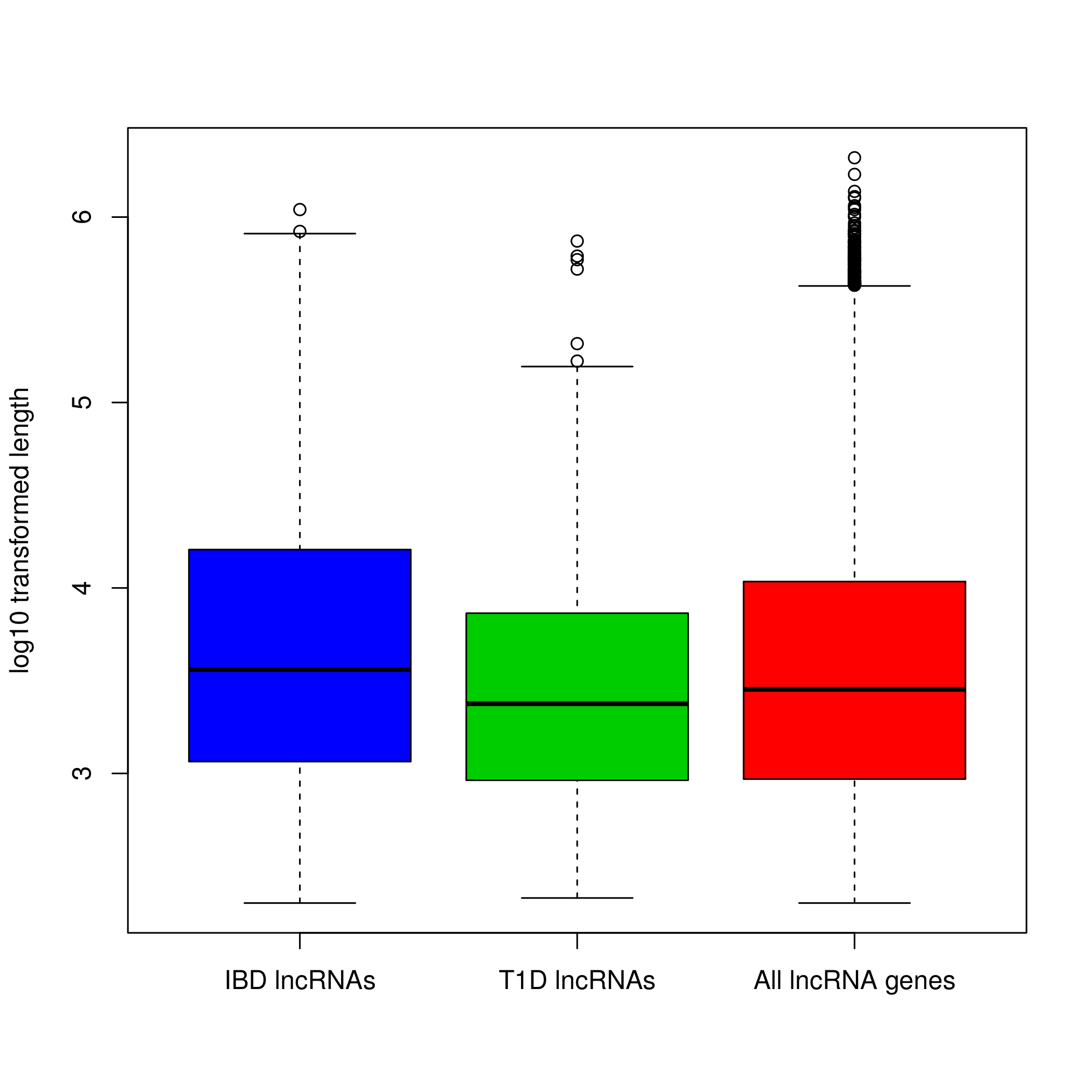

Supplement: Figure S2 — Comparison of length distribution of IBD and T1D loci-associated lncRNA genes. IBD and T1D loci-associated lncRNAs revealed significant differences in their average lengths as compared to the total lncRNAs. (TIFF) [file pone.0105723.s002.tiff]

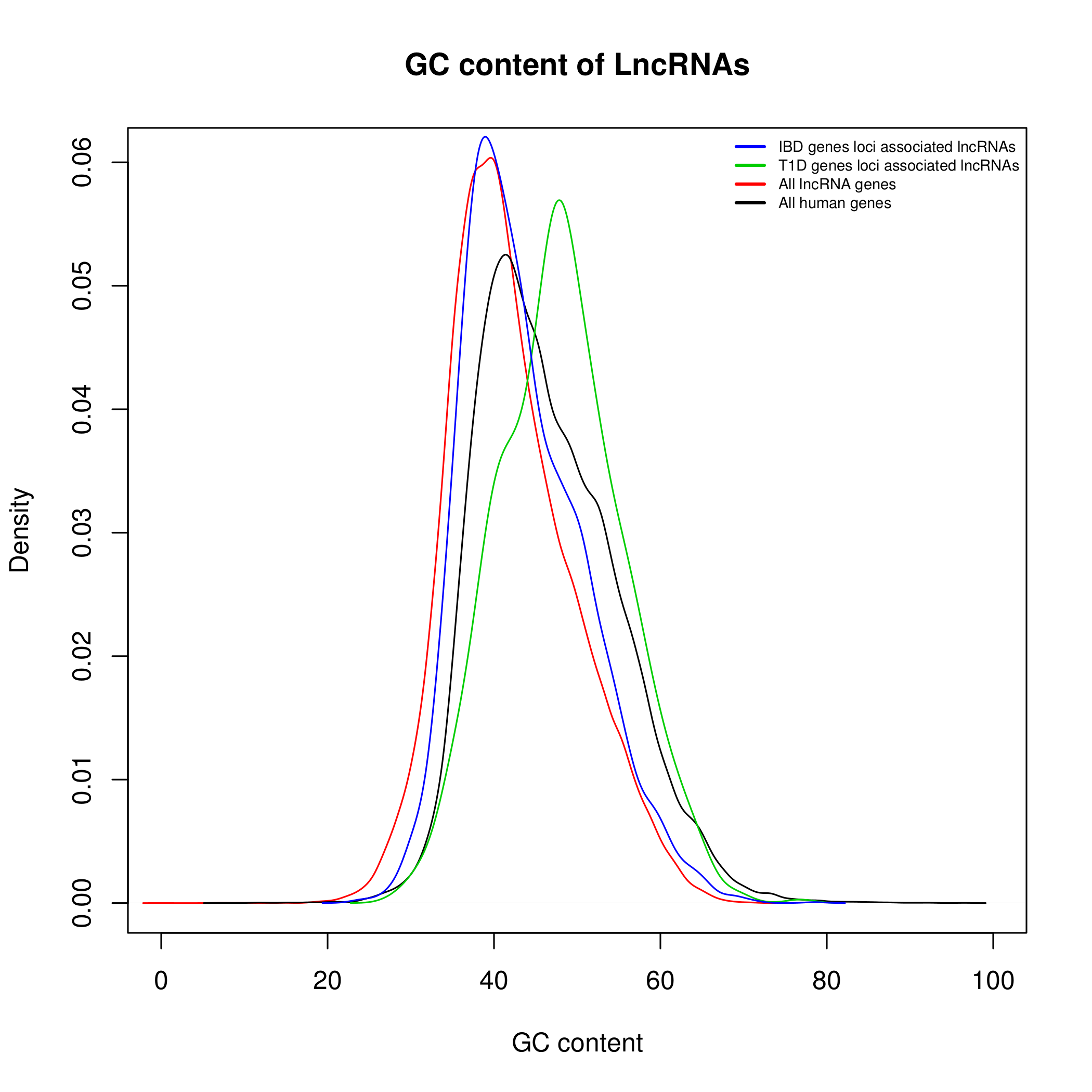

Supplement: Figure S3 — Comparison of GC content of IBD and T1D loci-associated lncRNA genes. Significantly higher GC content in T1D loci-associated lncRNA genes (p-value < 10e-6, Welch two sample t-test) was observed as compared to the background (total lncRNAs). (TIFF) [file pone.0105723.s003.tiff]

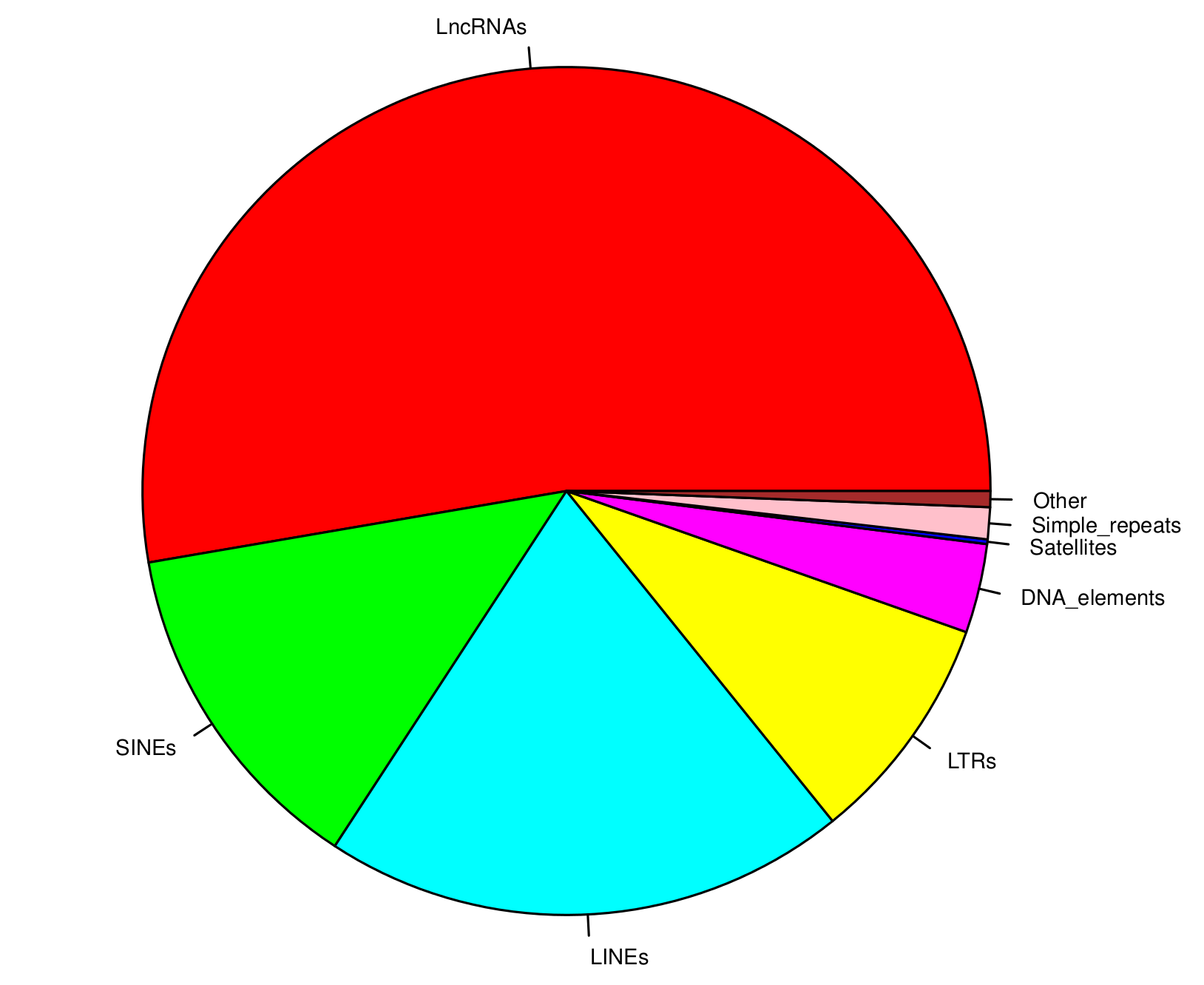

Supplement: Figure S4 — Distribution of repeat elements in Noncodev4 human lncRNA genes. (TIFF) [file pone.0105723.s004.tiff]

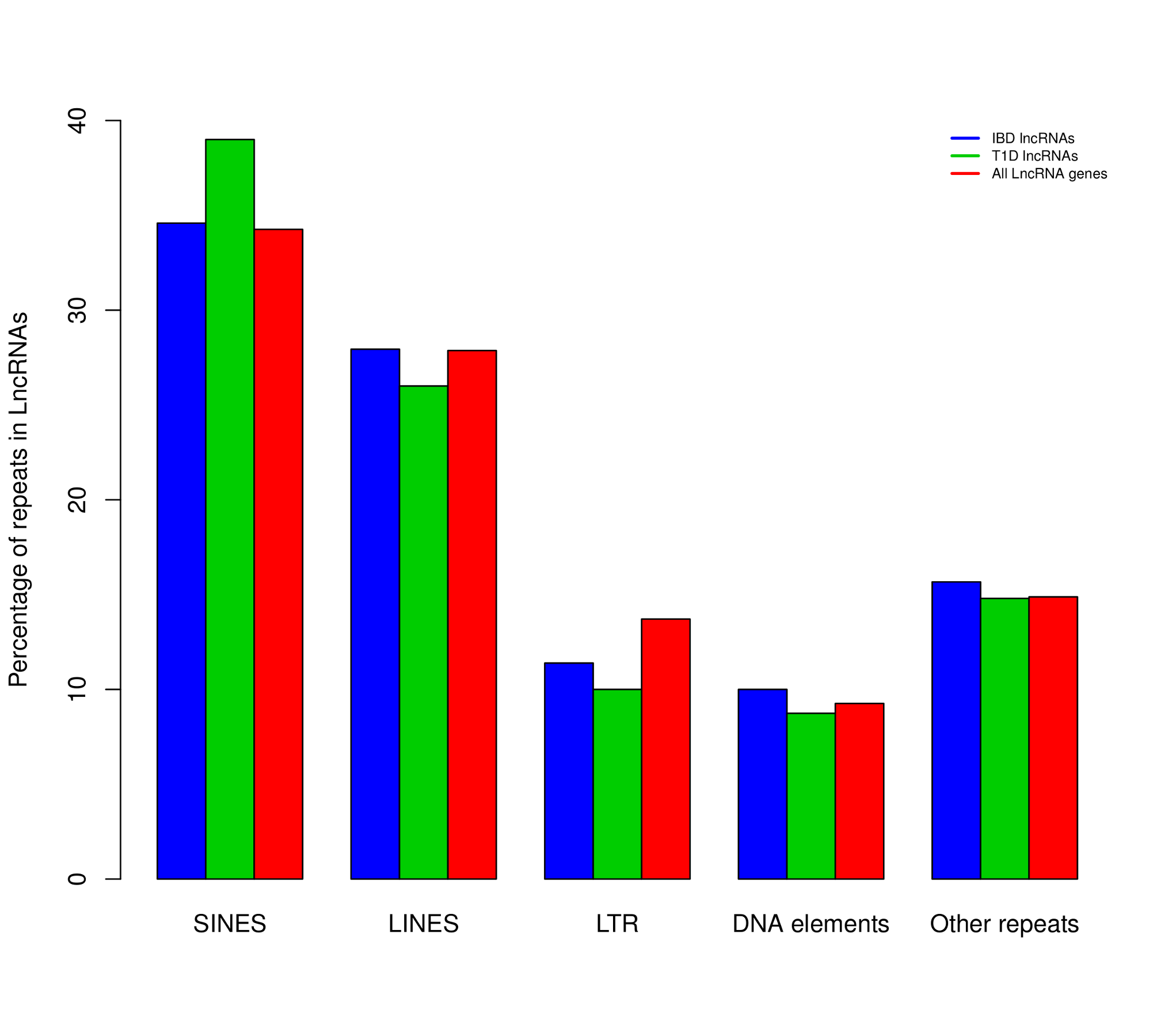

Supplement: Figure S5 — Relative abundance of interspersed repeat elements within IBD and T1D loci-associated lncRNA genes. Significant differences in the interspersed repeat element distributions for both IBD and T1D loci-associated lncRNAs were observed (p-value < 10e-6, Chi-square goodness of fit test) as compared to the background (total lncRNAs). (TIFF) [file pone.0105723.s005.tiff]

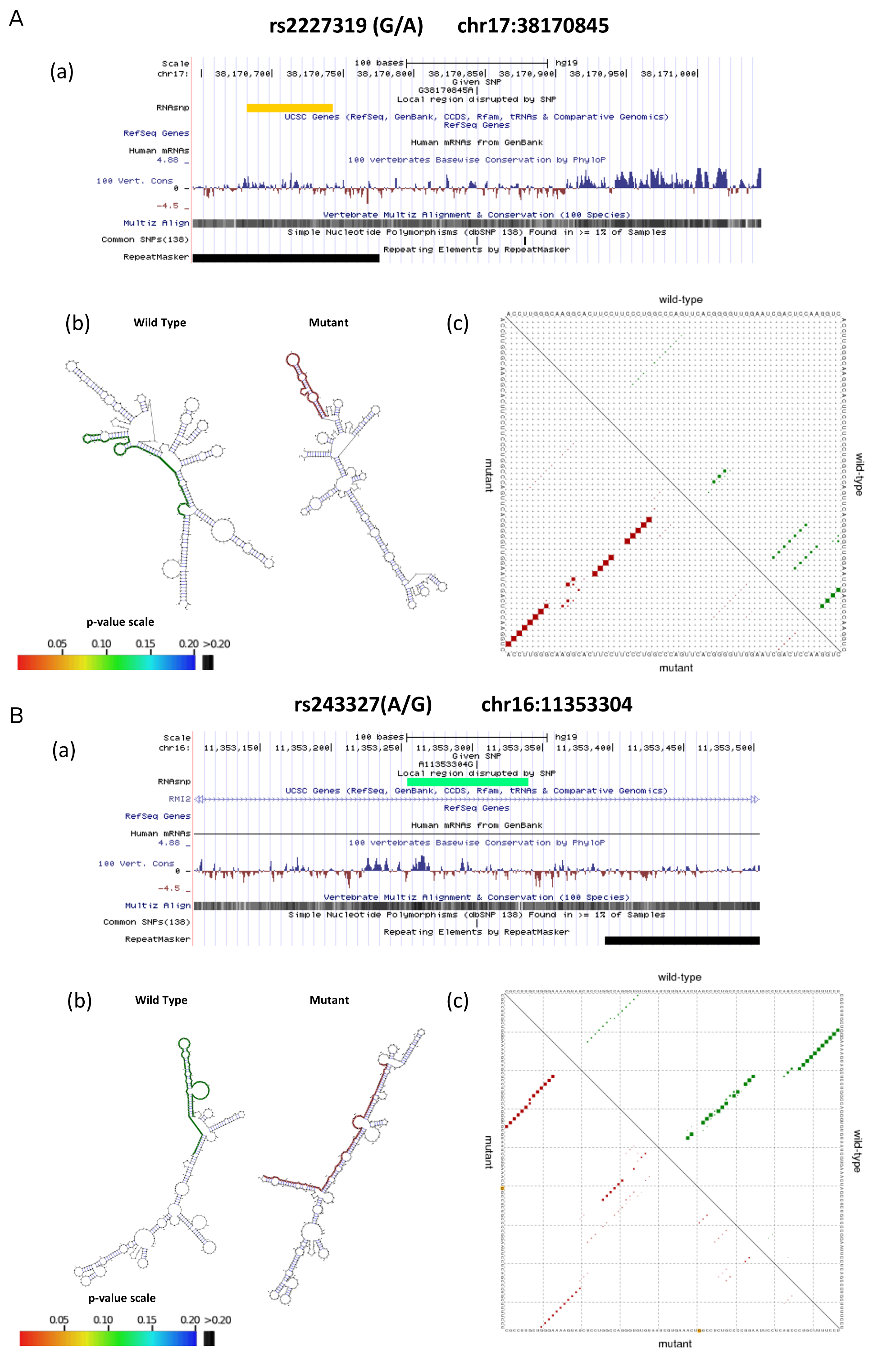

Supplement: Figure S6 — Top two structure-disruptive SNPs within IBD and T1D loci-associated lncRNAs (ranked based on RegulomeDB score and RNAsnp p-value). (A) SNP rs2227319 (structure-disruptive SNP within IBD loci-associated lncRNA NONHSAG021725). (B) SNP rs243327 (structure-disruptive SNP within T1D loci-associated lncRNA NONHSAG018599). (TIFF) [file pone.0105723.s006.tiff]

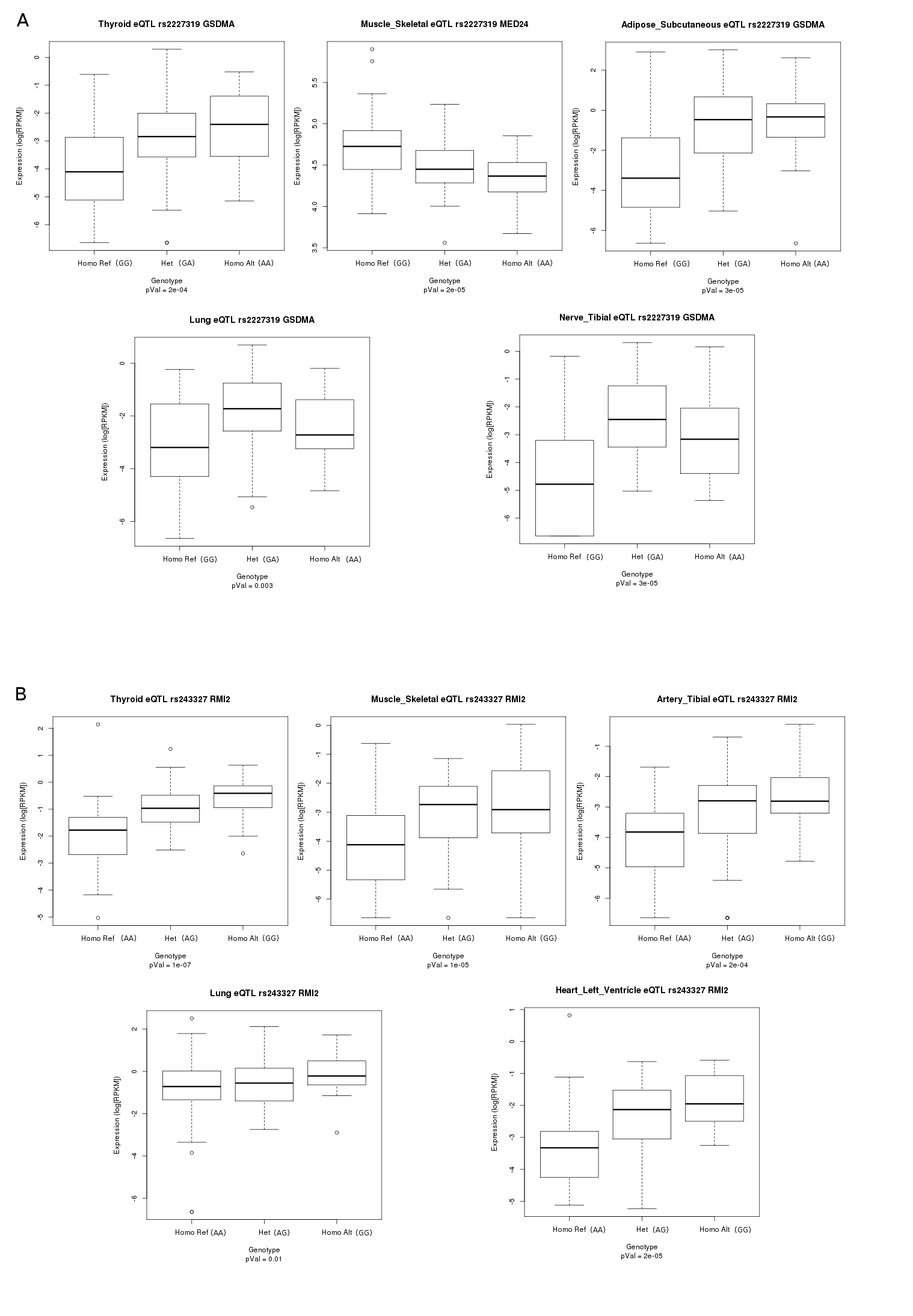

Supplement: Figure S7 — cis-eQTLs for the top two structure-disruptive SNPs within IBD and T1D loci-associated lncRNAs (ranked based on RegulomeDB score and RNAsnp p-value). (A) cis-eQTLs for structure-disruptive SNP rs2227319 within IBD loci-associated lncRNA NONHSAG021725. (B) cis-eQTLs for structure-disruptive SNP rs243327 within T1D loci-associated lncRNA NONHSAG018599. For each gene SNP association plot, p-values are displayed at the bottom. For a query SNP, GTEx provides pre-computed significant cis-eQTLs from 9 tissues (adipose subcutaneous, artery tibial, heart left ventricle, lung, muscle skeletal, nerve tibial, skin sun exposed, thyroid and whole blood) with more than 80 samples using a cis window of +/−1 MB around the transcription start site (TSS). (TIFF) [file pone.0105723.s007.tiff]

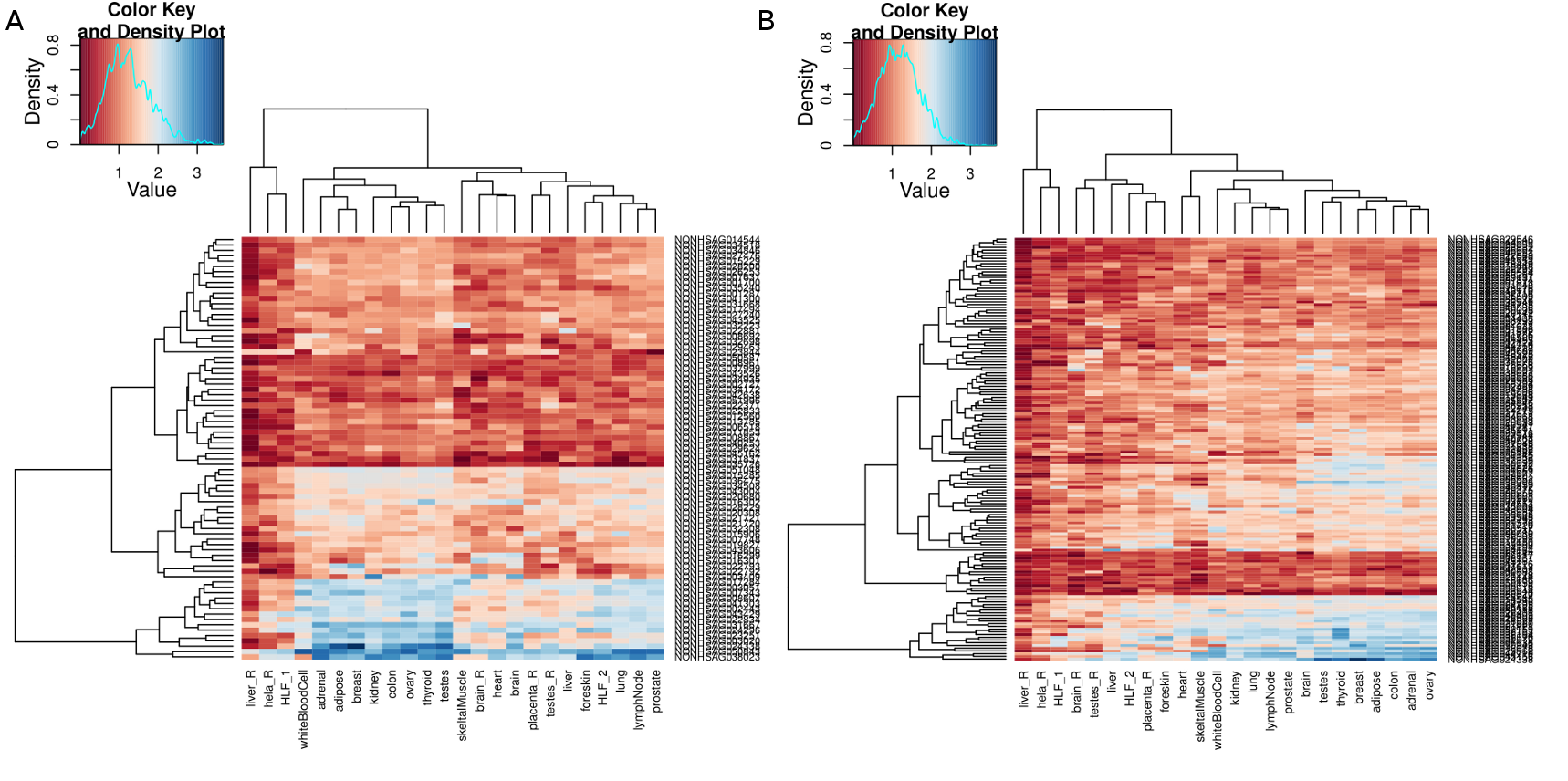

Supplement: Figure S8 — Expression levels for intergenic (A) and sense exonic/non-exonic (B) IBD loci-associated lncRNAs expressed across all HBM tissues (at FPKM threshold of >1). The FPKM threshold of >1 was used and the values were log10 transformed. (TIFF) [file pone.0105723.s008.tiff]

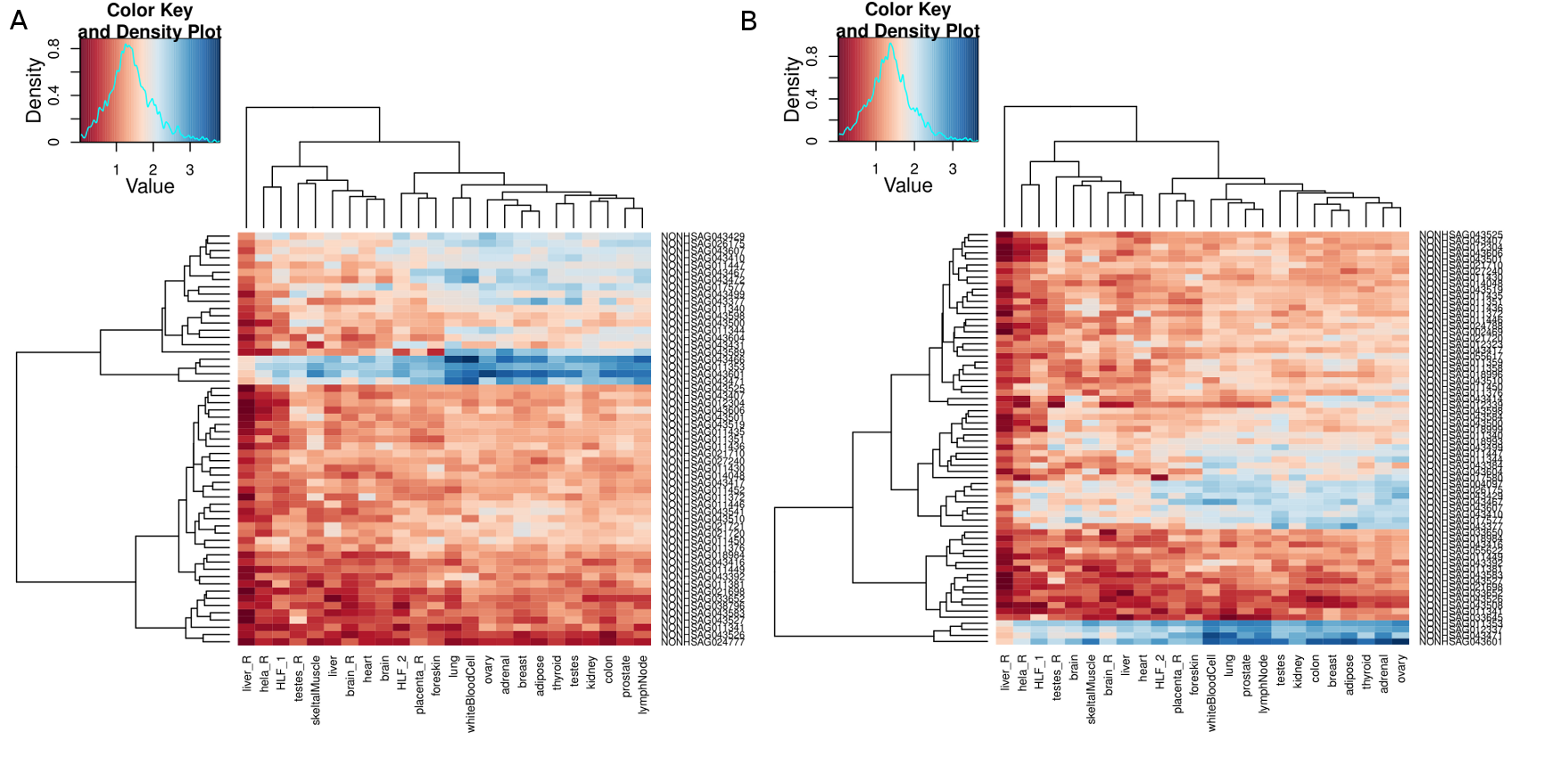

Supplement: Figure S9 — Expression levels for intergenic (A) and sense exonic/non-exonic (B) T1D loci-associated lncRNAs expressed across all HBM tissues (at FPKM threshold of >1). The FPKM threshold of >1 was used and the values were log10 transformed. (TIFF) [file pone.0105723.s009.tiff]

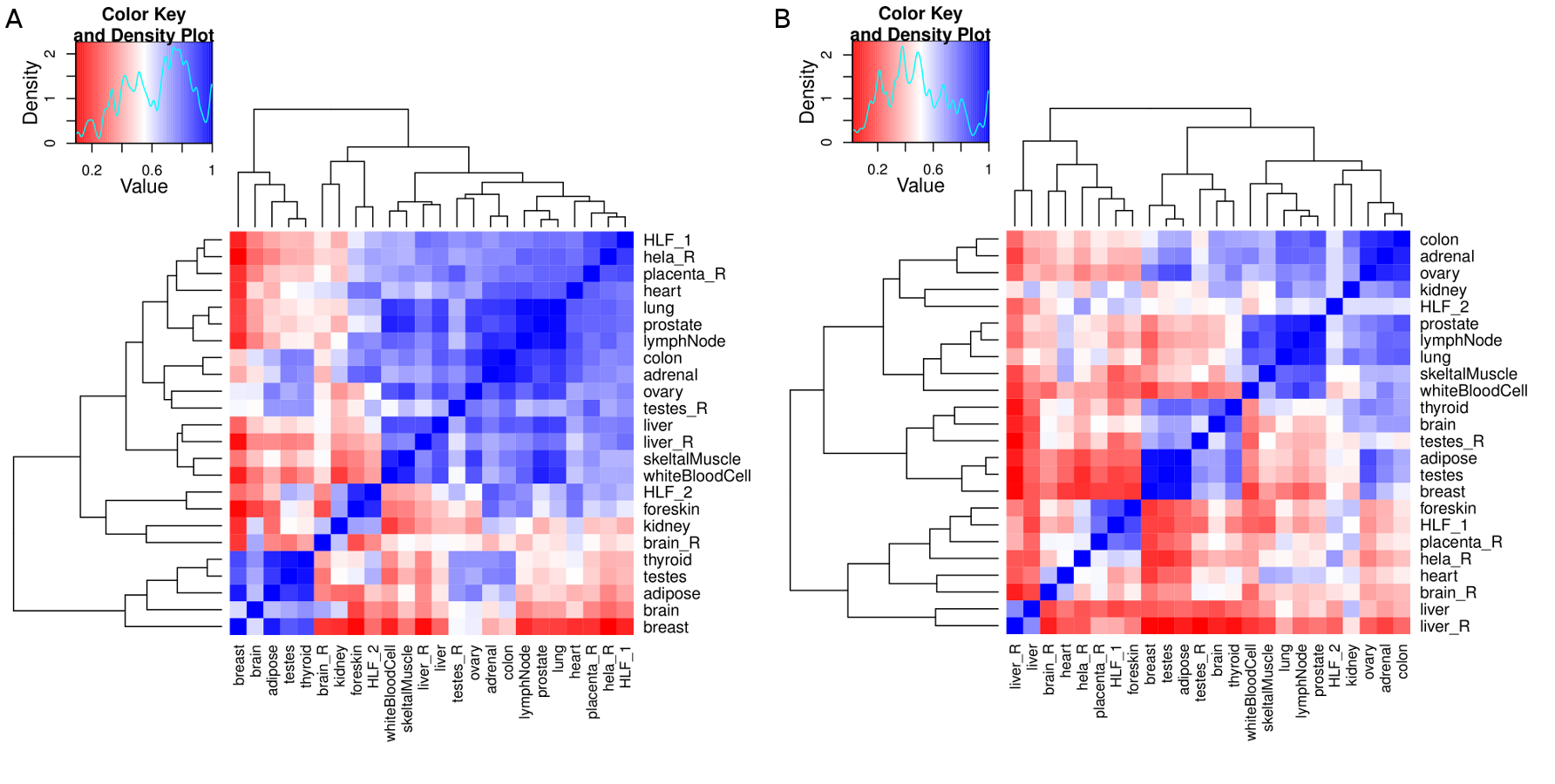

Supplement: Figure S10 — Correlation of expression for intergenic (A) and sense exonic/non-exonic (B) IBD loci-associated lncRNAs respectively expressed across all HBM tissues. The FPKM threshold of >1 was used and the values were log10 transformed. (TIFF) [file pone.0105723.s010.tiff]

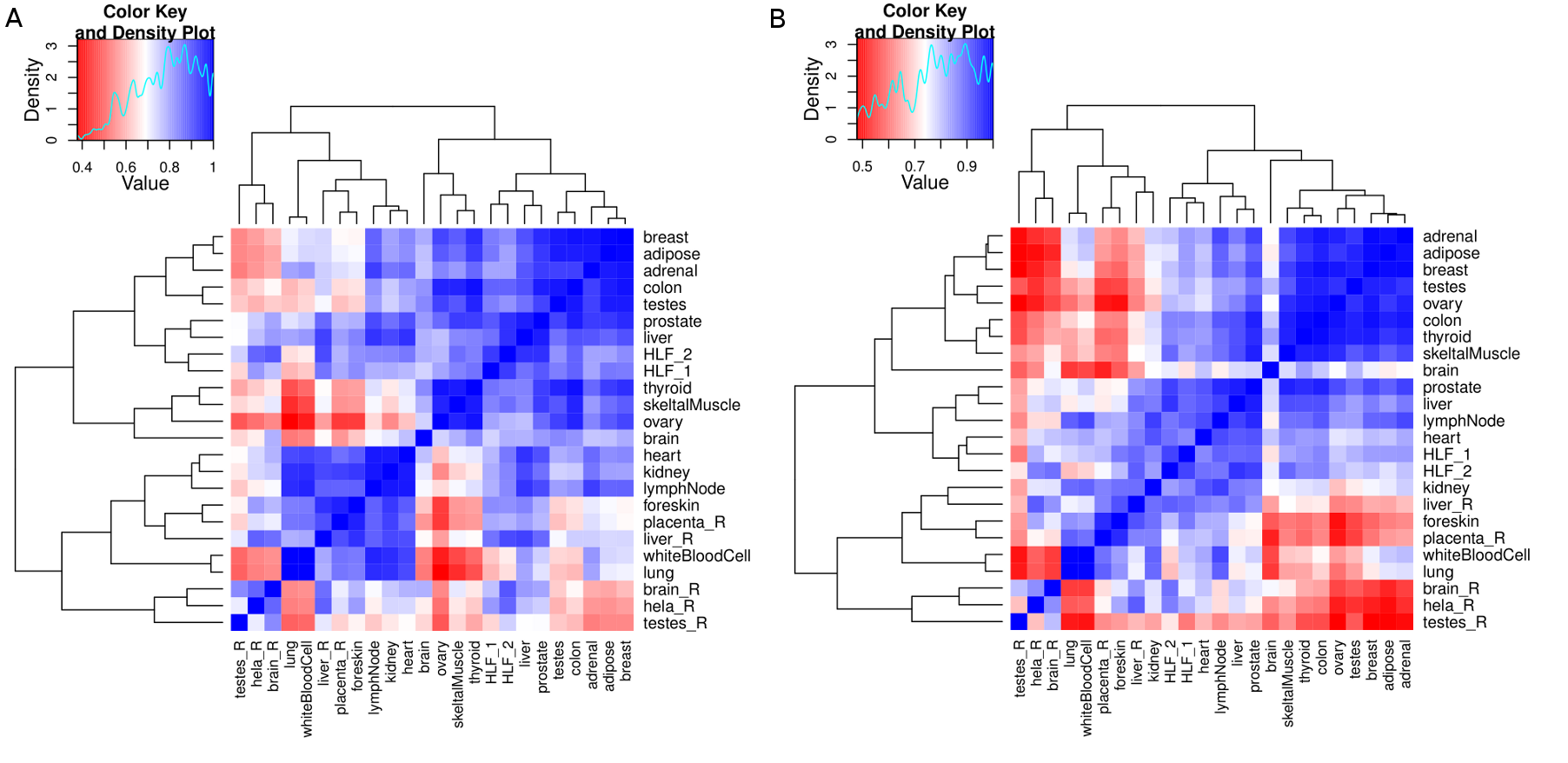

Supplement: Figure S11 — Correlation of expression for intergenic (A) and sense exonic/non-exonic (B) T1D loci-associated lncRNAs respectively expressed across all HBM tissues. The FPKM threshold of >1 was used and the values were log10 transformed. (TIFF) [file pone.0105723.s011.tiff]

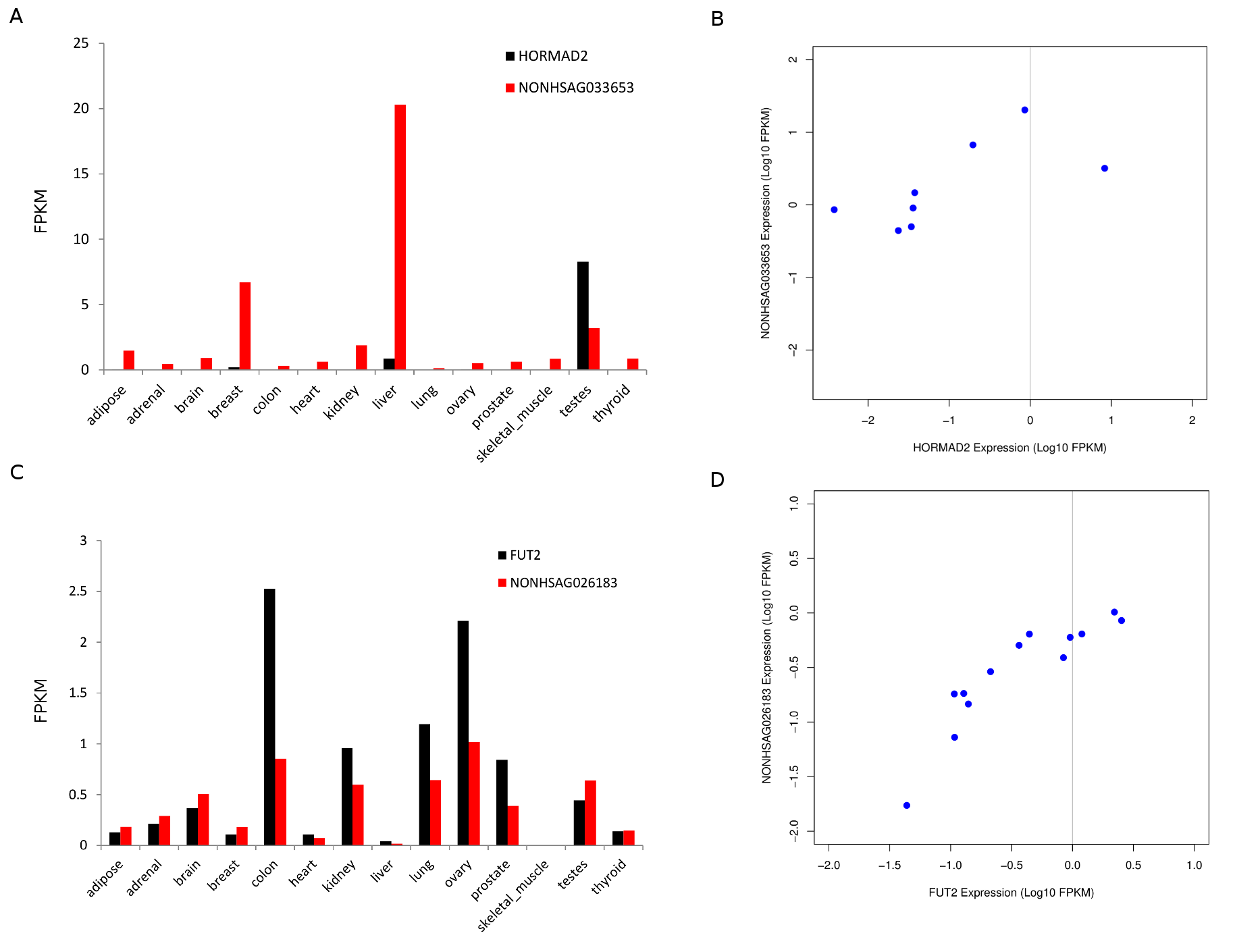

Supplement: Figure S12 — Tissue-specific gene expression profile of HORMAD2 (A) and FUT2 (C) candidate genes and their associated lncRNAs NONHSAG033653 and NONHSAG026183 across 14 tissues based on HBM data. Spearman correlations were calculated for lncRNAs NONHSAG033653 (B) and NONHSAG026183 (D) with HORMAD2 and FUT2 candidate genes respectively. Protein coding mRNA expression is plotted on the x-axis and lncRNA expression is shown on the y-axis both on log10 FPKM for 14 HBM tissues. (TIFF) [file pone.0105723.s012.tiff]
