# Supplementary material for: Effects of GWAS-Associated Genetic Variants on lncRNAs within IBD and T1D Candidate Loci
Source: PLoS One. 2014 Aug 21;9(8):e105723. doi: 10.1371/journal.pone.0105723 (PMC4140826; doi:10.1371/journal.pone.0105723)
Supplement: Table S2 — LncRNA expression profile of IBD and T1D loci-associated lncRNAs. Noncodev4 was used to retrieve expression data for sense exonic/non-exonic and antisense IBD and T1D loci-associated lncRNAs. Total number of expressed sense exonic/non-exonic and antisense lncRNAs was calculated using FPKM threshold >1. (DOCX) [file pone.0105723.s014.docx]

**Supplementary Table 2** LncRNA expression profile of IBD and T1D loci-associated lncRNAs

| **Tissues** | **4270 IBD lncRNAs (all)** | | **2132 IBD sense exonic/non-exonic lncRNAs** | | **1440 IBD antisense lncRNAs** | | **816 T1D lncRNAs (all)** | | **611 T1D sense exonic/non-exonic lncRNAs** | | **317 T1D antisense lncRNAs** | |
| --- | --- | --- | --- | --- | --- | --- | --- | --- | --- | --- | --- | --- |
|  | *Expressed* | *Not Expressed* | *Expressed* | *Not Expressed* | *Expressed* | *Not Expressed* | *Expressed* | *Not Expressed* | *Expressed* | *Not Expressed* | *Expressed* | *Not Expressed* |
| adipose | 1327 | 2943 | 865 | 1267 | 260 | 1180 | 326 | 490 | 277 | 334 | 131 | 186 |
| adrenal | 1518 | 2752 | 941 | 1191 | 326 | 1114 | 405 | 411 | 335 | 276 | 165 | 152 |
| brain | 1509 | 2761 | 948 | 1184 | 319 | 1121 | 344 | 472 | 282 | 329 | 139 | 178 |
| brain_R | 1066 | 3204 | 633 | 1499 | 235 | 1205 | 278 | 538 | 230 | 381 | 109 | 208 |
| breast | 1379 | 2891 | 882 | 1250 | 278 | 1162 | 353 | 463 | 301 | 310 | 144 | 173 |
| colon | 1322 | 2948 | 852 | 1280 | 268 | 1172 | 329 | 487 | 284 | 327 | 135 | 182 |
| foreskin | 982 | 3288 | 622 | 1510 | 193 | 1247 | 284 | 532 | 239 | 372 | 123 | 194 |
| heart | 1103 | 3167 | 707 | 1425 | 223 | 1217 | 283 | 533 | 244 | 367 | 119 | 198 |
| hela_R | 718 | 3552 | 460 | 1672 | 141 | 1299 | 208 | 608 | 179 | 432 | 89 | 228 |
| HLF_1 | 840 | 3430 | 546 | 1586 | 171 | 1269 | 225 | 591 | 192 | 419 | 95 | 222 |
| HLF_2 | 1053 | 3217 | 654 | 1478 | 236 | 1204 | 291 | 525 | 247 | 364 | 129 | 188 |
| kidney | 1330 | 2940 | 843 | 1289 | 259 | 1181 | 373 | 443 | 314 | 297 | 152 | 165 |
| liver | 873 | 3397 | 558 | 1574 | 169 | 1271 | 239 | 577 | 206 | 405 | 99 | 218 |
| liver_R | 454 | 3816 | 306 | 1826 | 76 | 1364 | 112 | 704 | 96 | 515 | 56 | 261 |
| lung | 1328 | 2942 | 864 | 1268 | 255 | 1185 | 361 | 455 | 306 | 305 | 145 | 172 |
| lymphNode | 1330 | 2940 | 837 | 1295 | 274 | 1166 | 380 | 436 | 318 | 293 | 157 | 160 |
| ovary | 1434 | 2836 | 911 | 1221 | 295 | 1145 | 378 | 438 | 319 | 292 | 153 | 164 |
| placenta_R | 1243 | 3027 | 811 | 1321 | 247 | 1193 | 333 | 483 | 286 | 325 | 137 | 180 |
| prostate | 1331 | 2939 | 849 | 1283 | 264 | 1176 | 355 | 461 | 301 | 310 | 149 | 168 |
| skeltalMuscle | 928 | 3342 | 598 | 1534 | 180 | 1260 | 275 | 541 | 232 | 379 | 114 | 203 |
| testes | 1782 | 2488 | 1055 | 1077 | 443 | 997 | 412 | 404 | 338 | 273 | 164 | 153 |
| testes_R | 1604 | 2666 | 859 | 1273 | 462 | 978 | 403 | 413 | 324 | 287 | 171 | 146 |
| thyroid | 1485 | 2785 | 937 | 1195 | 308 | 1132 | 375 | 441 | 313 | 298 | 154 | 163 |
| whiteBloodCell | 1147 | 3123 | 729 | 1403 | 231 | 1209 | 327 | 489 | 271 | 340 | 133 | 184 |
